# Structure–function analysis of tRNA t^6^A-catalysis, assembly, and thermostability of *Aquifex aeolicus* TsaD_2_B_2_ tetramer in complex with TsaE

**DOI:** 10.1016/j.jbc.2024.107962

**Published:** 2024-11-05

**Authors:** Shuze Lu, Mengqi Jin, Zhijiang Yu, Wenhua Zhang

**Affiliations:** School of Life Sciences, Key Laboratory of Cell Activities and Stress Adaptation of the Ministry of Education, Lanzhou University, Lanzhou, China

**Keywords:** *Aquifex aeolicus*, tRNA t^6^A, enzymatic reconstitution, crystal structure, TsaD_2_B_2_ tetramer, TsaD–TsaB–TsaE–tRNA assembly, ATP hydrolysis, oligomerization, GC base pairs content, thermostability

## Abstract

The universal *N*^6^-threonylcarbamoyladenosine (t^6^A) at position 37 of tRNAs is one of the core post-transcriptional modifications that are needed for promoting translational fidelity. In bacteria, TsaC uses *L*-threonine, bicarbonate, and ATP to generate an intermediate threonylcarbamoyladenylate (TC-AMP), of which the TC moiety is transferred to N6 atom of tRNA A37 to generate t^6^A by TsaD with the support of TsaB and TsaE. TsaD and TsaB form a TsaDB dimer to which tRNA and TsaE are competitively bound. The catalytic mechanism of TsaD and auxiliary roles of TsaB and TsaE remain to be fully elucidated. In this study, we reconstituted tRNA t^6^A biosynthesis using TsaC, TsaD, TsaB, and TsaE from *Aquifex aeolicus* and determined crystal structures of apo-form and ADP-bound form of TsaD_2_B_2_ tetramer. Our TsaD_2_B_2_–TsaE–tRNA model coupled with functional validations reveal that the binding of tRNA or TsaE to TsaDB is regulated by C-terminal tail of TsaB and a helical hairpin α1–α2 of TsaD. *A*. *aeolicus* TsaDB possesses a basal t^6^A catalytic activity that is stimulated by TsaE at the cost of ATP consumption. Our data suggest that the binding of TsaE to TsaDB induces conformational changes of α1, α2, α6, α7, and α8 of TsaD and C-terminal tail of TsaB, leading to the release of tRNA t^6^A and AMP. ATP-mediated binding of TsaE to TsaDB resets a t^6^A active conformation of TsaDB. Dimerization of TsaDB enhances thermostability and promotes t^6^A catalysis of TsaD_2_B_2_–tRNA, of which GC base pairs in anticodon stem are needed for the correct folding of thermophilic tRNA at higher temperatures.

The tertiary structures and decoding functions of tRNAs rely heavily on a large number of posttranscriptional modifications that are mainly found in anticodon stem loop, D-arm and T-arm of tRNAs (1, 2, 3, 4, 5). *N*^6^-threonylcarbamoyladenosine (t^6^A) is one of core modifications found uniquely at position 37 of tRNAs that decode ANN-codons (N being A, U, C, or G) in all the three domains of life (5, 6, 7, 8, 9). Structural and functional characterization revealed that t^6^A prevents an unwanted base-pairing between U33 and A37, stabilizes the configuration of anticodon stem loop, and enhances the anticodon–codon base pairing (10, 11, 12, 13). Consequently, the deficiency of tRNA t^6^A compromises translational fidelity (4, 14, 15, 16), leads to death of unicellular organisms (17, 18, 19), causes abnormal development of higher eukaryotes (15, 20), and is also implicated in human diseases, such as Galloway-Mowat syndrome (21, 22, 23, 24).

The biosynthesis of tRNA t^6^A is cooperatively accomplished by members of two conserved protein families—TsaC/Sua5/YRDC (COG0009) and TsaD/Kae1/Qri7/OSGEP (COG0533), and a number of organism-specific auxiliary proteins (9, 17, 18). In bacteria, tRNA t^6^A is biosynthesized by TsaC (YrdC), TsaD (YgjD), TsaB (YeaZ), and TsaE (YjeE), of which the latter three form an ATP-mediated TsaD–TsaB–TsaE complex (TsaDBE) (25, 26, 27, 28); in archaea and eukaryotic cytosols, tRNA t^6^A is biosynthesized by Sua5/YRDC and KEOPS complex (kinase, endopeptidase and other proteins of small size) that is composed of Kae1, Bud32, Cgi121, Pcc1, and Gon7 or Pcc2 (22, 29, 30, 31, 32, 33, 34, 35, 36); in eukaryotic mitochondrion, tRNA t^6^A is generated only by nuclear-encoded Sua5/YRDC and Qri7/OSGEPL1 (37, 38, 39). Biochemical analysis have determined that tRNA t^6^A biosynthesis proceeds in two consecutive steps (9, 40): in the first step, TsaC/Sua5/YRDC uses *L*-threonine, NaHCO_3_, and ATP to generate a short-lived intermediate threonylcarbamoyadenylate (TC-AMP) (26, 37, 41); in the second step, TsaDBE/KEOPS/Qri7/OSGEPL1 catalyzes transfer of TC-moiety from TC-AMP onto N6 atom of tRNA A37, leading to tRNA t^6^A (26, 28, 42). Bacterial tRNA t^6^A has been reconstituted *in vitro* using recombinant TsaC, TsaD, TsaB, and TsaE from mesophilic *Escherichia coli* and *Bacillus subtilis* (25, 26) and thermophilic *Thermotoga maritima* (28). The reaction scheme of tRNA t^6^A biosynthesis by TsaC, TsaD, TsaB, and TsaE is shown in Figure 1*A*. Luthra *et al*. determined that *T*. *maritima* TsaD_2_B_2_ catalyzes a single turnover of t^6^A-catalysis and subsequently turns into an inactive state, which is reactivated by TsaE at a cost of ATP (43). However, such a basal t^6^A-catalytic activity was not previously observed with neither *E*. *coli* TsaDB nor *B*. *subtilis* TsaDB (25, 26).

**Figure 1:**
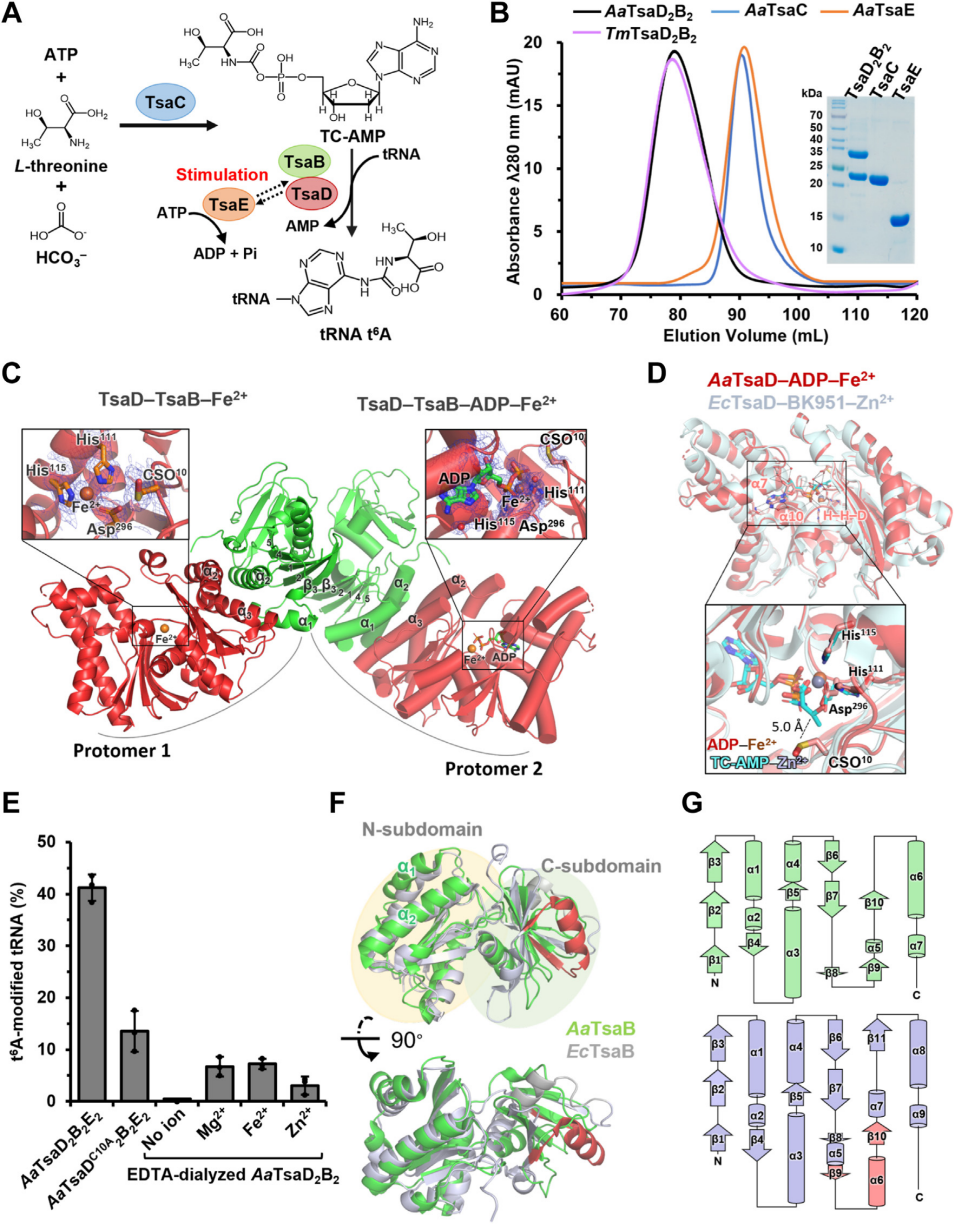
**Functional and structural characterization of TsaC, TsaD, TsaB, and TsaE from*****Aquifex******aeolicus* (*Aa*)**. *A*, reaction scheme of tRNA t^6^A biosynthesis by TsaC, TsaD, TsaB, and TsaE from bacteria. In the first step, TsaC protein uses *L*-threonine, HCO_3_^-^, and ATP to generate an unstable intermediate threonylcarbamoyladenylate (TC-AMP); in the second step, TsaDB complex transfers the TC-moiety from TC-AMP onto the N6 atom of tRNA A37, leading to tRNA t^6^A. The t^6^A-catalytic cycle of TsaDB is stimulated by TsaE at cost of ATP consumption. *B*, size-exclusion chromatography (SEC) profiles and SDS–PAGE analysis of *A*. *aeolicus* TsaD_2_B_2_, TsaC, TsaE, and *Thermotoga maritima* (*Tm*) TsaD_2_B_2_. *C*, crystal structure of *A*. *aeolicus* TsaD_2_B_2_ tetramer. TsaD and TsaB are shown in *red* and *green*, respectively. One TsaD–TsaB–Fe^2+^ protomer is cartooned as *helices* and the other TsaD–TsaB–ADP–Fe^2+^ protomer is cartooned as *cylindrical helices*. 2*F*_*o*_*–F*_*c*_ electron densities are contoured at 1.0 σ for ADP and Fe^2+^ in TsaD and for H^111^–H^115^–D^296^ motif and CSO^10^ of TsaD. *D*, structural juxtaposition of *A*. *aeolicus* TsaD–ADP–Fe^2+^ (colored in *red*) and *E*. *coli* (*Ec*) TsaD–BK951–Zn^2+^ (PDB: 6Z81, colored in *cyan*). The close-up view shows the binding orientations of ADP or TC-AMP (BK951) in the catalytic sites of TsaD. The *dashed line* denotes the distance from CSO^10^ to the threonyl-moiety of TC-AMP. *E*, t^6^A-modification efficiencies of 5 μM *A.aeolicus* TsaD_2_B_2_E_2_ and *A*. *aeolicus* TsaD^C10A^_2_B_2_E_2_ in combination with 5 μM TsaC toward 60 μM *in vitro* transcribed (IVT) *A*. *aeolicus* tRNA^Lys^_UUU_, and t^6^A-modification efficiencies of 5 μM EDTA-dialyzed TsaD_2_B_2_ toward 60 μM metal-deprived IVT *A*. *aeolicus* tRNA^Lys^_UUU_ in assays supplemented with 30 μM isolated TC-AMP and 100 μM MgCl_2_, FeCl_2_ or ZnCl_2_. Error bars represent standard deviations from triplicate measurements. *F*, structural juxtaposition of *A*. *aeolicus* TsaB and *E*. *coli* TsaB (PDB: 4YDU) manifests conserved N-terminal subdomains but varied C-terminal subdomains. The specific insertion segment (residues 143–162) of *E*. *coli* TsaB is highlighted in *red*. *G*, topological diagrams of *A*. *aeolicus* TsaB (*green*) and *E*. *coli* TsaB (*gray*). The secondary structures (β_9_α_6_β_10_) of the inserted segment of *E*. *coli* TsaB are shown in *red*. IVT, *in vitro* transcribed; PDB, Protein Data Bank; t^6^A, *N*^6^-threonylcarbamoyladenosine.

TsaD adopts an ancient ASKHA (acetate and sugar kinase/heat shock protein 70/actin) fold composed of two subdomains with a topology βββαβαβα (27, 44, 45), which is also typically conserved in structures of Kae1/Qri7 proteins (22, 30, 36, 37) and the TsaD-like domain of TsaN from *Pandoravirus salinus* (41). The t^6^A-catalytic site is formed between two subdomains of TsaD/Kae1/Qri7 proteins and binding of TC-AMP involves a metal-binding sphere that comprises an extremely conserved H–H–D motif (9). TsaB is a paralog of TsaD (18, 40). While the N-terminal subdomain of TsaB adopts a conserved topology of βββαβαβα, the C-terminal subdomain retains a reduced topology of ββαββα that does not support a nucleotide-binding site (46, 47, 48). Either TsaD or TsaB exists as a homodimer (27, 28). Equimolar mixture of TsaD and TsaB results in formation of a TsaDB dimer in *E*. *coli* and a TsaD_2_B_2_ tetramer in *T*. *maritima* (27, 28). TsaB homodimers adopt two different structural architectures: (1) mesophilic TsaB dimer is mediated by one pair of α1 and α2 from each monomer (27, 46, 47); (2) thermophilic *T*. *maritima* TsaB dimer is mediated by one pair of antiparallel β3 strands (43, 45, 48). Notably, the interacting interface of TsaDB comprises α2 and α3 of TsaD and α1 and α2 of TsaB (27, 44, 45), which form a conserved “helical bundle” found in dimers of TsaD–TsaD (27), TsaB–TsaB (27, 47), yeast Qri7–Qri7 (37), archaean Kae1–Pcc1 (30), plant KAE1–PCC1 (36) and human OSGEP–LAGE3 (22). At present, only a KEOPS–tRNA model was experimentally derived by combining the crystal structures of tRNA–Cgi121 (49) and archaean KEOPS subcomplex (30). According to the model, anticodon stem loop of tRNA is bound at the conserved “helical bundle” interface of Kae1 and Pcc1 (49, 50). In light of the structural conservation of TsaD/Kae1/Qri7 family proteins, the overall binding of tRNA to TsaD might conform to that of Kae1–tRNA and likely involves an interacting interface of TsaD and TsaB (9, 43, 50).

TsaE is composed of seven β-strands intertwined with three connecting α-helices on either side and adopts a fold that is characteristic of the small GTPases and some P-loop proteins (43, 51). TsaE is an intrinsically weak ATPase and forms an essential interaction network with TsaD and TsaB (52, 53). TsaE interacts with TsaDB dimer and stimulates the t^6^A-catalytic cycling of TsaDB (18, 26, 27, 43), which in turn activates the ATPase activity of TsaE (27, 43). Crystal structure of *T*. *maritima* TsaDBE complex revealed that TsaE is lodged at the interfacial region of TsaDB and directly contacts α1, α2, α4, and α6 of TsaD and C-terminal α7 of TsaB (43, 45). The ATP-binding site of TsaE interfaces with D158, K162, K166, and K213 of TsaD, which participate in stimulation of ATP to ADP hydrolysis by TsaE. Meanwhile, TsaE induces a flipping-out change of the helical hairpin of α1–α2 and creates an active conformation for binding of tRNA to TsaD. The TsaDBE structure coupled biochemical validation established a role of TsaE in resetting the t^6^A-catalytic activity of TsaDB (43).

Though mesophilic and thermophilic TsaDBs and TsaEs share remarkable structural and catalytic similarities (9), *T*. *maritima* TsaDB further dimerizes to form a TsaD_2_B_2_ tetramer that is capable of binding two molecules of TsaE (28). In-solution small-angle X-ray scattering (SAXS) analysis showed that only one molecule of tRNA is bound to one TsaDB protomer of *T*. *maritima* TsaD_2_B_2_ tetramer in a manner that is mutually exclusive to TsaE (43). It is unknown whether the oligomeric state of TsaD_2_B_2_ is commonly adopted by thermophilic organisms that grow optimally at above 60 °C (54). In particular, different molecular mechanisms underscoring mesophilic and thermophilic tRNA t^6^A biosynthetic systems remain to be elucidated. In this study, we reconstituted an *in vitro* enzymatic synthesis of tRNA t^6^A using recombinant TsaC, TsaD, TsaB, and TsaE from another thermophilic bacteria—*Aquifex aeolicus*. We determined crystal structures of *A*. *aeolicus* TsaD_2_B_2_ tetramer and characterized the assembly of TsaD_2_B_2_ in complex with tRNA and TsaE. Our results determined a functional and structural conservation of TsaDB dimer but different assembly and catalytic properties of TsaD_2_B_2_ tetramers. Furthermore, we demonstrate that the oligomerization of thermophilic TsaDB dimer and concentrated GC base pairs in anticodon stem of tRNAs enhance thermostability and t^6^A-catalytic activity of TsaD_2_B_2_–tRNA complex. These data and findings advance our understanding of the molecular workings of tRNA t^6^A-biosynthetic systems in bacteria.

## Results

### Structure of TsaD–TsaB tetramer from thermophilic *Aquifex aeolicus*

To gain more complete understanding of tRNA t^6^A biosynthetic systems from bacteria, we set out to characterize tRNA t^6^A-modifying enzymes from thermophilic *A*. *aeolicus*. Protein sequence alignments exhibit that *A*. *aeolicus* TsaD shares high identity to *E*. *coli* and *T*. *maritima* TsaD while TsaB and TsaE display relatively low sequence identities to their counterparts from *E*. *coli* and *T*. *maritima* (Fig. 1, *A–C*, Table 1), hinting variations in the functional and structural properties of TsaDB and TsaE. We purified recombinant TsaC, TsaD, TsaB, and TsaE (Fig. 1*B*) and performed crystallization screen of these proteins and tRNAs. We only obtained diffracting crystals of TsaD–TsaB alone and in the presence of ATP and MgCl_2_. We collected 2.0 Å resolution diffraction datasets using an in-house X-ray diffractor and determined two crystal structures of TsaD–TsaB by molecular replacement (55). The statistics of data collection, model building, and refinement are summarized in Table S1. Both crystals belonged to triclinic space group *P*3_1_2_1_ with almost identical unit cell dimensions (a = b = 97.72 Å and c = 132.20 Å). In sum, the two structures were well refined and completely modeled except for a short segment of TsaD (residues 220–225) and a short C-terminal tail of TsaB (residues 196–200). Inspection of electron densities confirmed presence of adenosine phosphates and a metal ion. Although proteins were crystallized in the presence of ATP, no electron density corresponding to the *γ*-phosphate was observed. This may be attributed to a lack of structural order of the *γ*-phosphate or the inherently weak ATPase activity of TsaD that hydrolyzes ATP to ADP. Hence, the observed density was modeled as ADP. The metal ion was modeled as Fe^2+^ based on two observations: (1) a Fe^2+^ was bound in corresponding place of *E*. *coli* TsaD (Protein Data Bank (PDB): 4YDU) and *Pyrococcus* *abyssi* Kae1 (PDB: 2IVP); (2) our assay in this study determined that FeCl_2_ confers the strongest t^6^A-stimulating effect on TsaD than ZnCl_2_ and MgCl_2_. Therefore, we resolved a crystal structure of *A*. *aeolicus* TsaD–TsaB in complex with ADP and Fe^2+^ and a crystal structure of *A*. *aeolicus* TsaD–TsaB in complex with Fe^2+^ (Fig. 1*C*). Moreover, we identified extra electron densities for side chains of Cys10 in both structures (Fig. 1*C*) and determined an oxidized state of Cys10, which was designated as CSO10 (56).

**Table 1:** RMSD values and sequence identities upon structural and sequence alignments of TsaD, TsaB, and TsaE proteins from *Aquifex aeolicus* (*Aa*), *Thermotoga maritima* (*Tm*), and *Escherichia coli* (*Ec*)

Target protein (PDB/AF ID)	Comparing protein (PDB/AF ID)	RMSD (Å)	Sequence identity (%)
*Aa*TsaD (8IFX)	*Tm*TsaD (6N9A)	1.814 (274 C_α_)	45
	*Ec*TsaD (6Z81)	0.889 (296 C_α_)	43
*Aa*TsaB (8IFX)	*Tm*TsaB (6N9A)	2.683 (108 C_α_)	18
	*Ec*TsaB (6Z81)	1.849 (78 C_α_)	15
*Aa*TsaE (O67011)	*Tm*TsaE (6N9A)	0.699 (83 C_α_)	32
	*Ec*TsaE (P0AF67)	0.921 (76 C_α_)	31
N-subdomain of *Aa*TsaB (8IFX) (residues 1–90 and 161–195)	N-subdomain of *Tm*TsaB (6N9A)	4.308 (84 C_α_)	--
	N-subdomain of *Ec*TsaB (6Z81)	1.814 (83 C_α_)	--
C-subdomain of *Aa*TsaB (8IFX) (residues 91–160)	C-subdomain of *Tm*TsaB (6N9A)	0.627 (10 C_α_)	--
	C-subdomain of *Ec*TsaB (6Z81)	7.529 (16 C_α_)	--

In both crystals, the asymmetrical unit contains a single TsaD and TsaB (Fig. S2*A*). α2 and α3 from TsaD and α1 and α2 from TsaB interact to form a typical “helical bundle” interacting interface of TsaDB dimer (Fig. 1*C*), which conforms to those of *E*. *coli* and *T*. *maritima* TsaDB dimers (Fig. S2*B*). Two TsaDB dimers from symmetrical units further interact and form a TsaD_2_B_2_ tetramer. Using crystallographic symmetry, we generated a model for TsaD_2_B_2_ tetramer in which two β3 strands of TsaBs interact in an antiparallel manner and lead to an extended β-sheet comprising β_5_β_4_β_1_β_2_β_3_ of TsaB_1_ and β_5_β_4_β_1_β_2_β_3_ of TsaB_2_ (Fig. 1*C*). The oligomeric state of such a TsaD_2_B_2_ tetramer is further confirmed by overlapped size-exclusion chromatography (SEC) profiles of *A*. *aeolicus* TsaD_2_B_2_ and *T*. *maritima* TsaD_2_B_2_ (Fig. 1*B*) (28).

### Configuration of the catalytic site of *A*. *aeolicus* TsaD

A persistent binding of adenine nucleotides or analogs of ATP (AMPPNP and ATP*γ*S) and divalent ions were observed in crystal structures of TsaD/Kae1/Qri7 proteins, such as *P*. *abyssi* Kae1–AMPPNP–Fe^2+^ (57), *E*. *coli* TsaD–ADP–Fe^2+^ (27), *Salmonella typhimurium* TsaD–ATP*γ*S–Zn^2+^ (44), *Saccharomyces cerevisiae* Qri7–AMP–Zn^2+^ (58) and *Homo sapiens* OSGEP–Mg^2+^ (22). However, these nucleotide-binding sites are supposed to bind the intermediate TC-AMP (Fig. 1*A*), as revealed in crystal structures of *E*. *coli* TsaD–BK951–Zn^2+^ (42) and *T*. *maritima* TsaD–carboxy-AMP–Zn^2+^ (43). Binding parameters suggest that AMPCPP—an analog of ATP—is more tightly bound to *E*. *coli* TsaD than BK951—an analogue of TC-AMP (27, 42). Hence, it remains unknown how abundant ATP is exchanged by TC-AMP for tRNA t^6^A-catalysis in cellular context. Our structures captured an apo-form of TsaDB and an ADP-bound form of TsaDB, which are perfectly superimposed with an RMSD value of 0.18 Å over 4017 atoms. The base of ADP is mainly stacked between α7 and α10 and β-phosphate is coordinated by a conserved metal-binding sphere that is composed of His^111^, His^115^, and Asp^296^ (Fig. 1*D*). Notably, ADP induces an inward movement of α6, α7, and α8 of TsaD (Fig. S2*A*), hinting that conformational changes of α7 could regulate the binding of adenine nucleotides. Superimposed structures of *A*. *aeolicus* TsaD–ADP and *E*. *coli* TsaD–TC-AMP (RMSD = 0.889 Å over 296 C_α_ atoms) manifest that the metal ion interacts directly with the carboxylate oxygens of TC-AMP and CSO10 is approximately 5 Å away from the threonyl-moiety of TC-AMP, hinting a participation of a metal ion and CSO10 in TC-AMP binding and/or t^6^A-catalysis (Fig. 1*D*).

To characterize the structure–function relationship, we set up a tRNA t^6^A modification assay (Fig. 1*A*), which contained 10 mM *L*-threonine, 15 mM NaHCO_3_, 5 mM ATP, 5 μM TsaC, 5 μM TsaD_2_B_2_ or 10 μM TsaDB, 10 μM TsaE, and 60 μM *in vitro* transcribed (IVT) tRNAs. The reaction was carried out at 37 °C and was stopped by heating at 98 °C. The reaction mixture was loaded on Urea–PAGE for separation of tRNAs which were subsequently extracted and digested into single nucleosides by nuclease P1 and alkaline phosphatase. Nucleosides were then applied to LC–MS for separation and analysis. The peak area of t^6^A was integrated and normalized to those of A, U, C, or G for calculation of t^6^A modification efficiency according to nucleotide sequence of tRNA and calibration using standard nucleosides. We first determined an efficient tRNA t^6^A-catalytic activity of TsaD_2_B_2_E_2_ (Figs. 3*D*, 1*E*). Structural superimposition shows that CSO10 of *A*. *aeolicus* TsaD is positioned in a close range to the threonylcarbamoyl-moiety of TC-AMP bound in *E*. *coli* TsaD (Fig. 1*D*), hinting a participation of CSO10 in TC-AMP binding and/or t^6^A-catalysis. Such a Cys10 is part of an ETSCD motif that is strictly conserved in bacterial TsaD proteins and mitochondrial Qri7/OSGEPL1 proteins (Fig. S2*C*). To test the functional involvement of Cys10, we mutated Cys10 to Ala and generated an *A*. *aeolicus* TsaD^C10A^_2_B_2_ variant, which behaved well over purification (Fig. S3*A*). The t^6^A-catalytic activity of TsaD^C10A^_2_B_2_E_2_ decreases by around 60% compared to that of TsaD_2_B_2_E_2_ (Fig. 1*E*), implying a direct participation of Cys10 or oxidization of Cys10 (CSO10) of TsaD in bacterial tRNA t^6^A modification.

In fact, the electron densities for the metal ions could be satisfactorily fitted with either a Fe^2+^ or a Zn^2+^, which participates in direct binding of ADP and TC-AMP (Fig. 1*D*). To test the role of metal ions in stimulating t^6^A-catalytic activity, we removed prebound metal ions in *A*. *aeolicus* TsaD_2_B_2_ *via* dialysis with EDTA. The SEC profile of EDTA-dialyzed TsaD_2_B_2_ was similar to that of untreated TsaD_2_B_2_ and shows an unaffected tertiary structure (Fig. S3*A*). In the metal-free assay, we only used EDTA-dialyzed TsaD_2_B_2_, HPLC-purified TC-AMP, and SEC-purified IVT tRNA^Lys^_UUU_. Our data show that EDTA-dialyzed *A*. *aeolicus* TsaD_2_B_2_ loses t^6^A-catalytic activity toward tRNA^Lys^_UUU_; adding MgCl_2_, FeCl_2_, or ZnCl_2_ restores of t^6^A-catalytic activity of TsaD_2_B_2_ (Figs. 3*E*, 1*E*). Low level of t^6^A modification was due to the exclusion of TsaE in the assay. Normalized t^6^A modification efficiencies manifest that MgCl_2_ and FeCl_2_ exhibit a potent stimulating effect on t^6^A-catalysis of TsaD while ZnCl_2_ conferred a lesser stimulating effect (Fig. 1*E*).

### *A*. *aeolicus* TsaB adopts a varied fold

Structural juxtaposition of *A*. *aeolicus* TsaB, *T*. *maritima* TsaB, and *E*. *coli* TsaB exhibit that *A*. *aeolicus* TsaB adopts a conserved fold of the N-subdomain and varied fold of the C-subdomain (Fig. S2*D*). In comparison to well-aligned TsaDs, alignments of TsaBs from *A*. *aeolicus*, *T*. *maritima* and *E*. *coli* gave large RMSD values over fewer C_α_ atoms (Table 1). Pair-wise structural alignment gave an RMSD value of 1.8 Å over 83 C_α_ atoms for the N-subdomains of *A*. *aeolicus* TsaB (residues 1–90 and 161–195) and *E*. *coli* TsaB. Only a small portion of backbone in the C-subdomain of *A*. *aeolicus* TsaB (residues 91–160) could be aligned to that of *E*. *coli* TsaB (Table 1). Analysis of structural topology revealed a varied distribution pattern of secondary structures of the C-subdomains of *A*. *aeolicus* TsaB and *E*. *coli* TsaB (Fig. 1, *F* and *G*). *A*. *aeolicus* TsaB is composed of more long-range unstructured regions (with well-defined electron densities) between β7 and α5. Of note, this varied fold of *A*. *aeolicus* TsaB was only correctly predicted by Alphafold2 (59), which provided us with a template for solving the crystal structure of *A*. *aeolicus* TsaD–TsaB by molecular replacement using MOLREP (55). *A*. *aeolicus* TsaB manifests a new variant of known fold of TsaB and adds value to structural analysis on molecular structural evolution (60). Sequence alignment reveals that *E*. *coli* TsaB has a 20 amino acids insertion compared to that of *A*. *aeolicus* and *T*. *maritima* (Fig. S1*B*). The inserted segment forms a βαβ structure (Fig. 1, *F* and *G*) that probably prevents formation of an extended antiparallel β-sheet as in TsaB–TsaB dimers from *A*. *aeolicus* or *T*. *maritima*.

### Dimerization of TsaDB promotes tRNA binding to TsaD_2_B_2_ and t^6^A-catalytic activity

**Figure 2:**
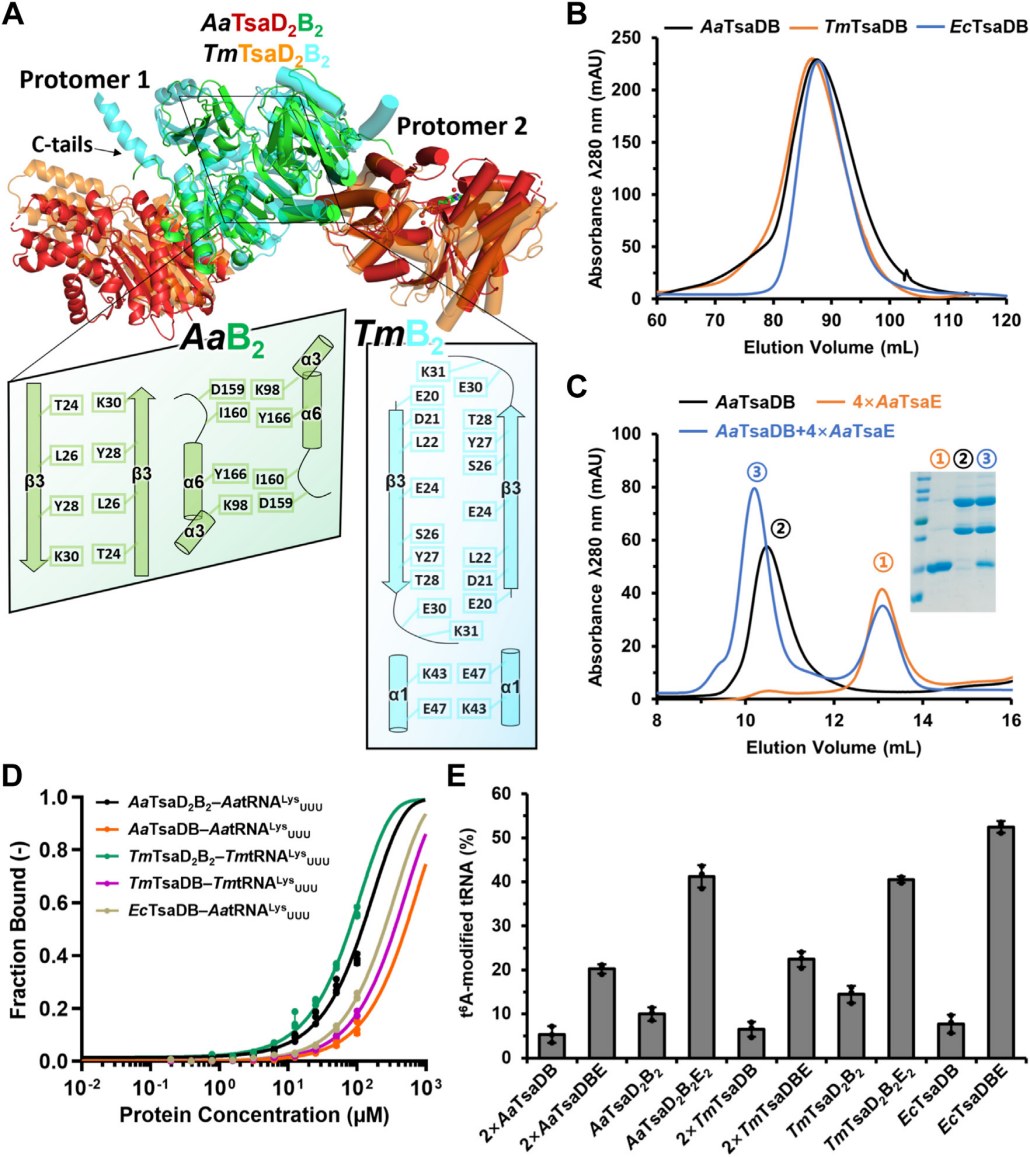
**Structural and functional characterization of TsaD**_**2**_**B**_**2**_**tetramer and TsaDB dimer**. *A*, structural juxtaposition of *Aa*TsaD_2_B_2_ and *Tm*TsaD_2_B_2_ (PDB: 6N9A) is labeled and shown in different colors. One TsaD–TsaB protomer is cartooned as *helices* and the other TsaD–TsaB protomer is cartooned as *cylindrical helices*. The close-up views show the interacting residues of *Aa*TsaB–TsaB and *Tm*TsaB–TsaB. *B*, SEC profiles of TsaDB dimers from *Aquifex aeolicus*, *Thermotoga maritima*, and *Escherichia coli*. *C*, SEC analysis of the interaction between *A*. *aeolicus* TsaDB and TsaE. Briefly, 400 μg TsaDB and 450 μg TsaE (1:4 in molar ratio) was applied to Superdex 75 10/300 GL column (GE HealthCare) and the inset shows SDS–PAGE analysis of proteins in fractions corresponding to the major peaks. *D*, microscale thermophoresis (MST) measurements of the interaction between 10 nM 5′ 6-FAM-tRNA^Lys^_UUU_ and 195 nM–100 μM TsaD_2_B_2_ tetramer or TsaDB dimer from *A*. *aeolicus*, *T*. *maritima* or *E*. *coli*. *E*, comparison of tRNA t^6^A modification efficiencies in complete assays using 5 μM TsaD_2_B_2_ or TsaD_2_B_2_E_2_ from *A*. *aeolicus* and *T*. *maritima*, 10 μM TsaDB or TsaDBE from *A*. *aeolicus* and *T*. *maritima*, and 5 μM TsaDB or TsaDBE from *E*. *coli*. Error bars represent standard deviations from triplicate measurements. t^6^A, *N*^6^-threonylcarbamoyladenosine; SEC, size-exclusion chromatography.

Crystal structures of *A*. *aeolicus* and *T*. *maritima* TsaD_2_B_2_ tetramer exhibit that two β3s of TsaB form two antiparallel β-strands that mainly support the dimerization of TsaDB (Fig. 2*A*). The dimerization interface also involves ionic bonds and hydrogen bonds between α3 and α6 (in *A*. *aeolicus* TsaD_2_B_2_) and ionic bonds between α1s (in *T*. *maritima* TsaD_2_B_2_). To test the t^6^A-catalytic activity of *A*. *aeolicus* and *T*. *maritima* TsaDB dimers, we made a series of mutations at the interacting interface of TsaB–TsaB. Briefly, *A*. *aeolicus* TsaDB variant was generated by mutating residues (E21A, K22A, V23A, T24A, F25A, L26A, H27A, Y28A, L29A, and K30A) of β3 in combination with mutating residues (K98A, D159A, I160A, and Y166A) of α3 and α6 while *T*. *maritima* TsaDB variant was rather simply generated by mutating residues (E20A, D21A, L22A, E24A, S26A, Y27A, T28A, E30A, and K31A) of β3. Hereafter, we use TsaD_2_B_2_ and TsaDB to indicate a TsaD–TsaB–TsaB–TsaD tetramer and a TsaD–TsaB dimer variant, respectively. SEC profiles of *A*. *aeolicus* and *T*. *maritima* TsaDB overlap with that of *E*. *coli* TsaDB dimer (Figs 2*B*, S3*B*), demonstrating that these mutations completely disrupt the homodimerization of TsaDB.

In crystal structure of *T*. *maritima* TsaDBE (43, 45), TsaE form 15 and 9 hydrogen bonds with TsaD and TsaB, respectively (Table S2). Structural model coupled sequence alignment shows that only eight and seven pairs of interacting residues between TsaE and TsaD are conserved in *A*. *aeolicus* TsaDBE and *E*. *coli* TsaDBE, respectively. No conserved pairs of interacting residues between TsaE and TsaB are found in *A*. *aeolicus* TsaDBE and *E*. *coli* TsaDBE (Table S2). Native gel interaction analysis shows that *T*. *maritima* TsaDB readily interacts with TsaE, irrespective of ATP (Fig. S4*A*). In contrast, *A*. *aeolicus* TsaDB interacts with TsaE only in the presence of ATP (Fig. S4*A*). SEC and SDS–PAGE analyses have confirmed the formation of *A*. *aeolicus* TsaDBE ternary complex in the presence of ATP (Fig. 2*C*). Using electrophoresis mobility shift assay (EMSA), we observed no binding of *A*. *aeolicus* tRNA^Lys^_UUU_ to *A*. *aeolicus* TsaDB (Fig. S4*F*) but a weak binding to *A*. *aeolicus* TsaD_2_B_2_ (Fig. S4*G*). Under same conditions, a binding of *E*. *coli* tRNA^Lys^_UUU_ to *E*. *coli* TsaDB was not observed (Fig. S4*H*). In contrast, *T*. *maritima* tRNA^Lys^_UUU_ is strongly bound to *T*. *maritima* TsaDB (Fig. S4*I*) and TsaD_2_B_2_ (Fig. S4*J*), with a dissociation constant (*K*_d_) of 1.72 μM and 1.20 μM (Fig. S4*M*), respectively. We used microscale thermophoresis (MST) to compare the binding affinities of tRNAs to TsaDB and TsaD_2_B_2_ by incubating 10 nM 5′ 6-FAM-tRNA^Lys^_UUU_ with a serial dilution of 100 μM proteins (Fig. S5). A saturated binding was not achieved even at a molar ratio of 10,000, which did not permit an accurate calculation of the *K*_d_ value (Fig. 2*D*). Nevertheless, shifts of the binding points in MST profile demonstrate that *A*. *aeolicus* and *T*. *maritima* TsaD_2_B_2_ tetramers possess higher binding affinities toward their cognate tRNA^Lys^_UUU_s than their TsaDB dimers and *E*. *coli* TsaDB dimer (Fig. 2*D*).

**Figure 3:**
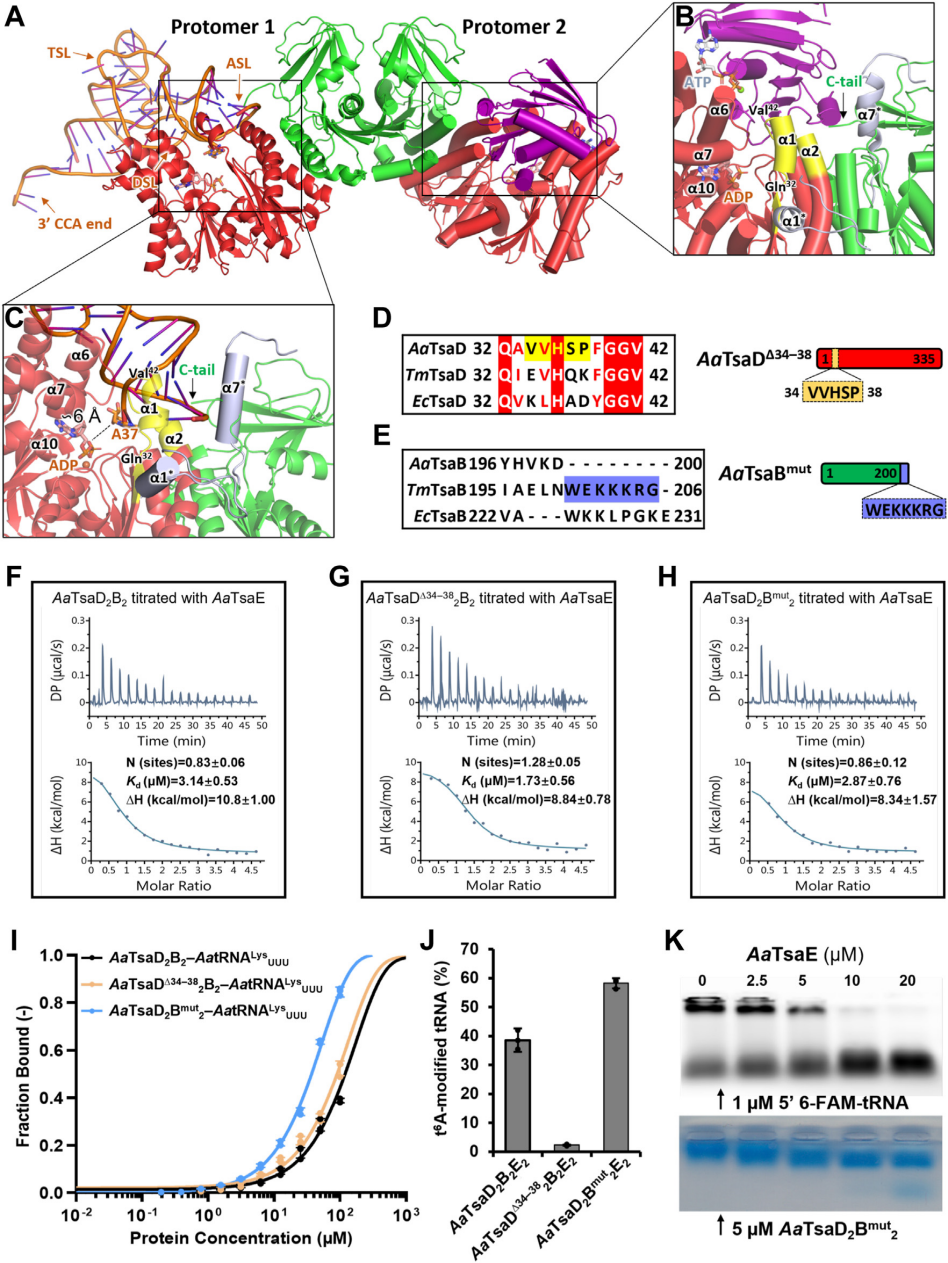
**Structural model of *Aquifexaeolicus* TsaD**_**2**_**B**_**2**_**in complex with TsaE or tRNA and functional validations**. *A*, a structural model for *A*. *aeolicus* TsaD_2_B_2_–TsaE–tRNA complex. tRNA is bound to one TsaDB protomer and TsaE is bound to the other TsaDB protomer. *B*, a close view of the assemble of *A*. *aeolicus* TsaE onto TsaDB. *C*, a close view of the binding of anticodon stem loop of tRNA in the catalytic center of TsaD. The helical hairpin α1–α2 of *A*. *aeolicus* TsaD are shown in *yellow*; the equivalent α1 of *Thermotoga maritima* TsaD and C-terminal α7 of *T*. *maritima* TsaB are shown in *gray* and indicated with *stars*. The other parts of *T*. *maritima* TsaDB are omitted for clarity. *D*, local sequence alignment of residues 32 to 42 of TsaDs from *A*. *aeolicus*, *T*. *maritima*, and *E*. *coli*, and the schematic representation of *A*. *aeolicus* TsaD^Δ34–38^ mutant. *E*, local sequence alignment of the C-terminal tail of TsaBs from *A*. *aeolicus*, *T*. *maritima*, and *E*. *coli*, and the schematic representation of *A*. *aeolicus* TsaB^mut^ mutant. *F*–*H*, ITC analysis of the interaction between *A*. *aeolicus* TsaD_2_B_2_ (F), TsaD^Δ34–38^_2_B_2_ (G) or TsaD_2_B^mut^_2_ (H) and TsaE. Representative plots from an ITC experiment are shown with raw data in the upper panel and curve fit in the lower panel. *I*, MST measurements of the interaction between 10 nM 5′ 6-FAM-tRNA^Lys^_UUU_ and 195 nM–100 μM TsaD_2_B_2_, TsaD^Δ34–38^_2_B_2_ or TsaD_2_B^mut^_2_. *J*, quantification of tRNA t^6^A modification efficiencies of 5 μM *A.aeolicus* TsaD_2_B_2_E_2_, TsaD^Δ34–38^_2_B_2_E_2_ or TsaD_2_B^mut^_2_E_2_ toward 60 μM IVT tRNA^Lys^_UUU_. Error bars represent standard deviations from triplicate measurements. *K*, EMSA analysis of the competition binding of TsaE and tRNA to TsaD_2_B^mut^_2_. TsaE at indicated concentration was added to 5 μM *Aa*TsaD_2_B^mut^_2_ that was preincubated with 1 μM 5′ 6-FAM-tRNA^Lys^_UUU_. The *upper* panel shows the presence of 5′ 6-FAM-tRNA^Lys^_UUU_ and *bottom* panel shows the presence of *Aa*TsaD_2_B^mut^_2_E_2_ and unbound TsaE. IVT, *in vitro* transcribed; 6-FAM, 6-carboxyfluorescein; ITC, isothermal titration calorimetry; MST, microscale thermophoresis; t^6^A, *N*^6^-threonylcarbamoyladenosine; EMSA, electrophoresis mobility shift assay.

Luthra *et al*. reported that *T*. *maritima* TsaD_2_B_2_ possesses a basal catalytic activity and is capable of catalyzing a single turnover of t^6^A-catalysis (43). However, such a basal t^6^A-catalytic activity was not previously observed with neither *E*. *coli* TsaDB (25) nor *B*. *subtilis* TsaDB (26). We set up an *in vitro* assay using 5 μM TsaD_2_B_2_ or 10 μM TsaDB and 60 μM SEC-purified tRNAs^Lys^_UUU_, and tested tRNA t^6^A modification efficiencies of TsaDB dimers and TsaD_2_B_2_ tetramers from *A*. *aeolicus*, *T*. *maritima*, and *E*. *coli*. Altogether, we determined that all these TsaDB dimers possess basal t^6^A-catalytic activities toward their cognate tRNAs^Lys^_UUU_ (Figs. 2*E*, S3*G*). Remarkably, *A*. *aeolicus* TsaD_2_B_2_ and *T*. *maritima* TsaD_2_B_2_ exhibit higher t^6^A-catalytic activities than their two-fold TsaDB dimers (in molar concentration). Accordingly, the t^6^A-catalytic efficiency of TsaD_2_B_2_E_2_ has doubled compared to that of two-fold TsaDBE (Figs. 3*F*, 2*E*). These data suggest that dimerization of TsaDB dimer enhances the binding of tRNAs^Lys^_UUU_ to TsaD_2_B_2_ (Fig. 2*D*) and promotes t^6^A-catalytic efficiency of TsaD_2_B_2_ (Fig. 2*E*).

### Docked model of *A*. *aeolicus* TsaD_2_B_2_–TsaE–tRNA

We retrieved a prediction model for *A*. *aeolicus* TsaE (AF-O67011-F1) from AlphaFold database. Sequence and structural alignments show that *A*. *aeolicus* TsaE is highly identical to *T*. *maritima* TsaE and *E*. *coli* TsaE, except for a C-terminal helix that is absent in *A*. *aeolicus* TsaE (Figs. S1*C*, S2*E*). We cloned and purified recombinant TsaEs from *A*. *aeolicus*, *T*. *maritima*, and *E*. *coli* (Fig. S3*C*). In SEC, *A*. *aeolicus* TsaE elutes out at a volume larger than those of monomeric TsaEs from *T*. *maritima* and *E*. *coli* (27, 28), demonstrating that *A*. *aeolicus* TsaE exists as a monomer. These recombinant TsaEs are all active in stimulating the t^6^A-catalytic activity of TsaDB and TsaD_2_B_2_ (Fig. 2*E*). Our native gel analysis shows an association between *A*. *aeolicus* TsaE and *T*. *maritima* TsaDB in the presence of ATP (Fig. S4*B*), demonstrating a structural similarity of *A*. *aeolicus* TsaE and *T*. *maritima* TsaE. We aligned our crystal structure of *A*. *aeolicus* TsaDB and Alphafold model of *A*. *aeolicus* TsaE to crystal structure of *T*. *maritima* TsaDBE ternary complex (45) and generated a structural model for *A*. *aeolicus* TsaDBE complex (Fig. S2*F*). Likewise, we aligned *A*. *aeolicus* TsaDB to archaean KEOPS–tRNA complex (49) and generated a model for *A*. *aeolicus* TsaDB–tRNA complex (Fig. S2*G*), in which the binding orientation of tRNA conforms to the SAXS model of *T*. *maritima* TsaD_2_B_2_–tRNA (43). Based on our crystal structure of *A*. *aeolicus* TsaD_2_B_2_ tetramer (Fig. 1*C*), we combined models of *A*. *aeolicus* TsaDB–TsaE and TsaDB–tRNA and generated a model for *A*. *aeolicus* TsaD_2_B_2_–TsaE–tRNA complex (Fig. 3*A*). According to the model, TsaD_2_B_2_ could simultaneously bind one TsaE and one tRNA, which are bound to the same TsaDB protomer in a mutually exclusive manner. The model of TsaD_2_B_2_–TsaE–tRNA hints that the helical hairpin α1–α2 of TsaD and the C-terminal tail of TsaB are involved in regulating the binding of TsaE and tRNA to TsaDB.

### α1 of TsaD is required for tRNA t^6^A catalysis by TsaDBE

In our crystal structure of *A*. *aeolicus* TsaDB, the helical hairpin α1–α2 (Ser31–Glu50) of TsaD simultaneously contacts the connecting loop of α6 and α7 of TsaD and the C-terminal tail of TsaB (Fig. 3*B*), and adopts a conformation that prevents anticodon stem loop of tRNA from docking into the t^6^A-catalytic site of TsaD (Fig. 3*C*). In contrast, the equivalent α1–α2 segment (Ser31–His50) of *T*. *maritima* TsaD undergoes a flipping-out conformational change and only retains α1 in crystal structure of *T*. *maritima* TsaDBE (Figs. 2*F*, 3*B*). To characterize the role of α1–α2, we disrupted α1 of *A*. *aeolicus* TsaD by chopping off five residues—^34^VVHSP^38^—and generated a TsaD^Δ34–38^ mutant (Fig. 3*D*), which behaved well over purification and still forms a stable TsaD^Δ34–38^_2_B_2_ tetramer in SEC (Fig. S3*A*). Our interaction analysis by native gel shows that *A*. *aeolicus* TsaD^Δ34–38^_2_B_2_ still does not interact with TsaE in the absence of ATP (Fig. S4*C*). Noticeably, more fraction of TsaE is bound to *A*. *aeolicus* TsaD^Δ34–38^_2_B_2_ compared to TsaD_2_B_2_ (Fig. S4*E*). We determined by isothermal titration calorimetry (ITC) that, in the presence of AMPPNP, *A*. *aeolicus* TsaD_2_B_2_ binds to TsaE with a *K*_d_ value of 3.14 μM (Fig. 3*F*) while TsaD^Δ34–38^_2_B_2_ binds to TsaE with a *K*_d_ value of 1.73 μM (Fig. 3*G*), which is consistent with the native gel analysis. EMSA analysis shows that the interaction between *A*. *aeolicus* tRNA^Lys^_UUU_ and *A*. *aeolicus* TsaD^Δ34–38^_2_B_2_ (Fig. S4*K*) is tighter than that between *A*. *aeolicus* tRNA^Lys^_UUU_ and TsaD_2_B_2_ (Fig. S4*G*). MST measurements exhibit that *A*. *aeolicus* TsaD^Δ34–38^_2_B_2_ binds tRNA^Lys^_UUU_ stronger than *A*. *aeolicus* TsaD_2_B_2_ (Fig. 3*I*). Interestingly, only a small amount of t^6^A (2.8% t^6^A-modified tRNA^Lys^_UUU_) was detected by LC–MS in assay using *A*. *aeolicus* TsaD^Δ34–38^_2_B_2_E_2_ and tRNA^Lys^_UUU_ (Fig. 3*J*, S3*H*), whose t^6^A-catalytic activity is substantially lower than that of *A*. *aeolicus* TsaD_2_B_2_ (10% t^6^A-modified tRNA^Lys^_UUU_, Fig. 2*E*). These data suggest that the deletion of α1 leads to an open conformation of α2 (Fig. 3*B*), which favors the binding of TsaE and tRNA but impedes tRNA t^6^A catalysis.

### C-terminal tail of TsaB promotes tRNA binding and t^6^A-catalytic activity of TsaD_2_B_2_

**Figure 4:**
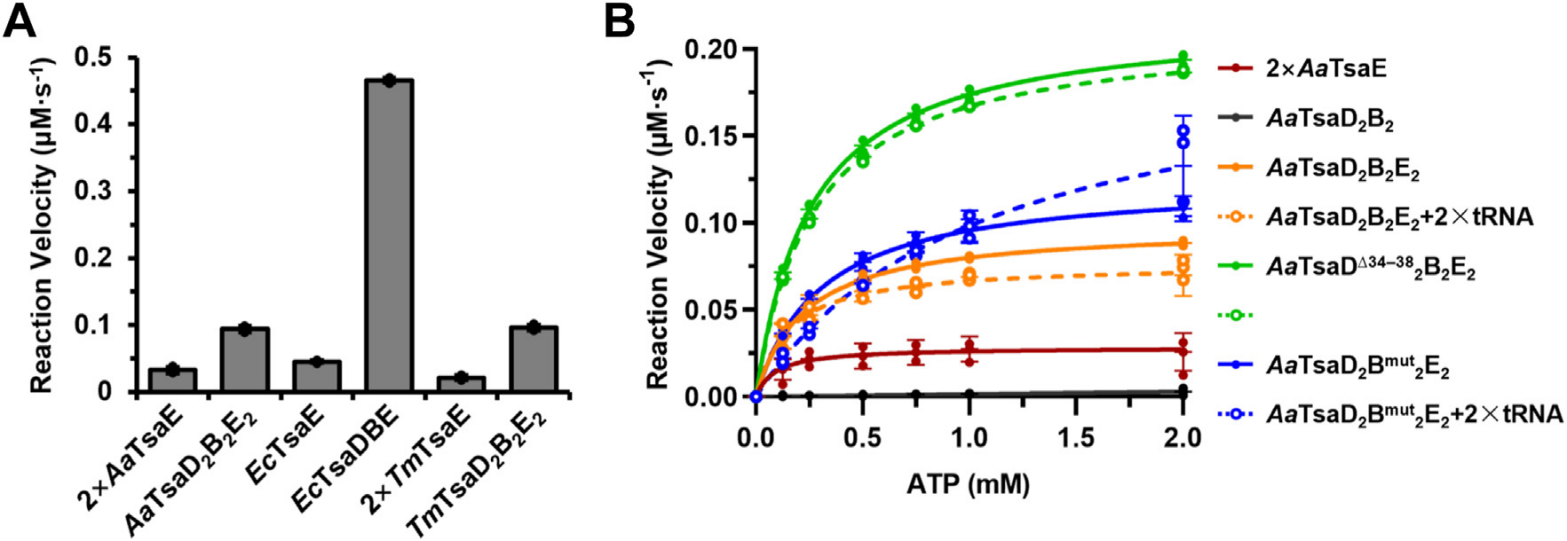
**Analysis of ATPase activity of TsaE in the presence of TsaDB and tRNA**. *A*, initial velocities of 4 mM ATP to ADP hydrolysis by 10 μM *A*. *aeolicus* TsaE, 5 μM *A.aeolicus* TsaD_2_B_2_E_2_, 5 μM *E.coli* TsaE, 5 μM *E.coli* TsaDBE, 10 μM *T*. *maritima* TsaE, or 5 μM *T.maritima* TsaD_2_B_2_E_2_. Error bars represent standard deviations from triplicate measurements. *B*, steady-state kinetics analysis of the ATPase activity of 10 μM *A.aeolicus* TsaE in the presence of 5 μM *A.aeolicus* TsaD_2_B_2_ or mutants and 10 μM IVT tRNA^Lys^_UUU_. Kinetic parameters are summarized in Table 2. Error bars represent standard deviations from triplicate measurements. IVT, *in vitro* transcribed

Structural comparison and sequence alignment show that the C-terminal tails of TsaB from *A*. *aeolicus*, *E*. *coli*, and *T*. *maritima* are not strictly conserved (Fig. 2*A*, S2*B*). The C-tail of *T*. *maritima* TsaB is rich in positively charged residues and adopts a long α-helix (α7) that directly interact with TsaE (Fig. 3*B*). Deletion of C-tail (Leu189–Gly206) of *T*. *maritima* TsaB leads to a complete dissociation of tRNA from TsaD_2_B_2_ (43). In contrast, the C-tail of *A*. *aeolicus* TsaB is seven amino acids shorter and adopts no secondary structures (Fig. 3, *B* and *E*). The model of *A*. *aeolicus* TsaDB–TsaE–TsaDB–tRNA shows that the C tail of TsaB protrudes toward tRNA and likely interacts with bound tRNA. Based on the sequence alignment (Fig. 3*E*), we grafted the C tail (^200^WEKKKRG^206^) of *T*. *maritima* TsaB to *A*. *aeolicus* TsaB (Fig. 3*E*) and generated a “gain-of-function” mutant of *A*. *aeolicus* TsaB^201WEKKKRG207^—TsaB^mut^. SEC profiles demonstrate that *A*. *aeolicus* TsaB^mut^ and TsaD form a stable TsaD_2_B^mut^_2_ tetramer (Fig. S3*A*). Native gel analysis shows that TsaD_2_B^mut^_2_ still does not interact with TsaE in the absence of ATP (Fig. 4, *D* and *E*). But the ATP-mediated interaction between TsaD_2_B^mut^_2_ and TsaE is comparable to that of TsaD_2_B_2_ and TsaE, as analyzed by ITC (Fig. 3, *F* and *H*). Thus, the grafted C tail does not seem to promote the binding of TsaE to TsaDB *via* electrostatic interactions (Fig. 3*B*) (45). Remarkably, EMSA analysis shows that *A*. *aeolicus* tRNA^Lys^_UUU_ is almost completely bound to TsaD_2_B^mut^_2_ at a molar ratio of 1:8 (*K*_d_ ≈ 1.16 μM) (Fig. 4, *L* and *M*) whereas only a fraction of tRNA^Lys^_UUU_ is bound to TsaD_2_B_2_ at a molar ratio of 100 (Fig. S4*G*). We also determined by MST that *A*. *aeolicu*s TsaD_2_B^mut^_2_ has a higher binding affinity toward tRNA^Lys^_UUU_ than *A*. *aeolicu*s TsaD_2_B_2_ (Fig. 3*I*). These data demonstrate that *A*. *aeolicus* TsaD_2_B^mut^_2_ acquired an enhanced tRNA-binding ability which is attributed to the grafted C-tail. Quantification of t^6^A-modified tRNA^Lys^_UUU_ shows that the t^6^A-catalytic efficiency of TsaD_2_B^mut^_2_E_2_ has increased by 25% compared to that of TsaD_2_B_2_E_2_ (Fig. 3*J*, S3*H*).

### Communication between tRNA t^6^A catalysis by TsaDB and ATP hydrolysis by TsaE

Central to mechanistic understanding of bacterial tRNA t^6^A biosynthetic systems is to elucidate the communications between t^6^A-catalysis by TsaDB and ATP hydrolysis-mediated reactivation of TsaDB by TsaE (Fig. 1*A*). Luthra *et al*. determined that *T*. *maritima* TsaD_2_B_2_ catalyzes a single turnover of tRNA t^6^A-catalysis and then switches into a catalytically inactive state, which could be reactivated by TsaE *via* an ATP to ADP hydrolysis-based mechanism (43). The requirement of TsaE and an extra consumption of ATP (not being substrate for TC-AMP) were documented for *E*. *coli* TsaDB (25, 27) and *B*. *subtilis* TsaDB (26). We have determined that *A*. *aeolicus* TsaD_2_B_2_ or TsaDB is also capable of generating tRNA t^6^A and adding equimolar TsaE potently stimulates the t^6^A-catalytic efficiency (Fig. 2*E*). Using an in-solution NADH–coupled ATPase assay, we show that hydrolysis of ATP to ADP by TsaE is stimulated by TsaDBs, of which *E*. *coli* TsaDB confers a markedly strong stimulating effect while *A*. *aeolicus* or *T*. *maritima* TsaD_2_B_2_ shows a lesser effect (Fig. 4*A*). Our competition-binding assay shows that adding equimolar *A*. *aeolicus* TsaE to TsaD_2_B^mut^_2_–tRNA^Lys^_UUU_ complex leads to complete release of tRNA^Lys^_UUU_ (Fig. 3*K*), demonstrating that TsaDB possesses a higher binding affinity toward TsaE than tRNA^Lys^_UUU_.

To analyze the complex interactions among TsaD_2_B_2_, TsaE, and tRNA, we measured steady-state kinetic parameters of ATP hydrolysis by TsaE in the presence of TsaD_2_B_2_ and tRNA (Fig. 4*B* and Table 2). *A*. *aeolicus* TsaE possesses an intrinsically weak activity in hydrolyzing ATP to ADP at a low rate and adding TsaD_2_B_2_ increases the maximum velocity (V_max_) from 0.037 to 0.098 μM·s^-1^ but also increases the Michaelis–Menten constant (*K*_m_) from 0.096 to 0.228 mM. As a consequence, the catalytic efficiency (*k*_cat_/*K*_m_) values for TsaE (0.078 mM^-1^ s^-1^) and TsaD_2_B_2_E_2_ (0.086 mM^-1^ s^-1^) are comparable. Further addition of tRNA^Lys^_UUU_ to TsaD_2_B_2_E_2_ reduces both V_max_ (0.075 μM s^-1^) and *K*_m_ (0.157 mM), resulting in a comparable *k*_cat_/*K*_m_ value (0.095 μM s^-1^). Remarkedly, addition of TsaD^Δ34–38^_2_B_2_ leads to a robust increase of V_max_ (0.219 μM s^-1^) and *k*_cat_/*K*_m_ (0.170 μM s^-1^). In contrast, adding tRNA to TsaD^Δ34–38^_2_B_2_E_2_ does not affect V_max_ (0.211 μM s^-1^) and *k*_cat_/*K*_m_ (0.159 μM s^-1^). In comparison to TsaD_2_B_2_, adding TsaD_2_B^mut^_2_ slightly increases V_max_ (0.125 μM s^-1^) and *K*_m_ (0.304 mM), leading to a very similar *k*_cat_/*K*_m_ (0.086 mM^-1^ s^-1^). Interestingly, adding tRNA to TsaD_2_B^mut^_2_E_2_ potently increases V_max_ (0.201 μM s^-1^) and *K*_m_ (1.05 mM), and drastically reduces *k*_cat_/*K*_m_ (0.038 mM^-1^ s^-1^). These kinetic parameters coupled interaction analysis reveal that α6 and α8 of TsaD directly contacts the switch II of TsaE and stimulates the turnover of ATP to ADP (Figs. 2*F*, 4*A*); binding of tRNA to TsaDB frees ADP-bound TsaE and speeds up exchange of ATP-bound TsaE; enhanced binding of TsaE to TsaD^Δ34–38^_2_B_2_ promotes the turnover of ATP to ADP; enhanced binding of tRNA to TsaD_2_B^mut^_2_ stimulates the initial reaction velocity but slows down the exchange of ATP-bound TsaE, leading to a low level of catalytic efficiency.

**Table 2:** Summary of the steady-state kinetic parameters of ATP to ADP hydrolysis by *A*. *aeolicus* TsaE in the presence of *A*. *aeolicus* TsaD_2_B_2_, TsaD^Δ34–38^_2_B_2_, TsaD_2_B^mut^_2_, and *A*. *aeolicus* tRNA^Lys^_UUU_

Proteins	V_max_ (μM·s^-1^)	*K*_m_ (mM)	*k*_cat_ (s^-1^)	*k*_cat_/*K*_m_ (mM^-1^ s^-1^)
2 × *Aa*TsaE	0.0374	0.096 ± 0.0449	0.007 ± 0.0007	0.078
TsaD_2_B_2_	Not determined			
TsaD_2_B_2_E_2_	0.0982	0.228 ± 0.0479	0.020 ± 0.0012	0.086
TsaD_2_B_2_E_2_+2 × tRNA	0.0746	0.157 ± 0.0654	0.015 ± 0.0017	0.095
TsaD^Δ34–38^_2_B_2_E_2_	0.2187	0.258 ± 0.0223	0.044 ± 0.0011	0.170
TsaD^Δ34–38^_2_B_2_E_2_+2 × tRNA	0.2114	0.266 ± 0.0032	0.042 ± 0.0002	0.159
TsaD_2_B^mut^_2_E_2_	0.1247	0.304 ± 0.0577	0.025 ± 0.0015	0.082
TsaD_2_B^mut^_2_E_2_+2 × tRNA	0.2015	1.049 ± 0.4023	0.040 ± 0.0082	0.038

### Oligomerization of TsaDB promotes thermostability and t^6^A-catalytic activity

**Figure 5:**
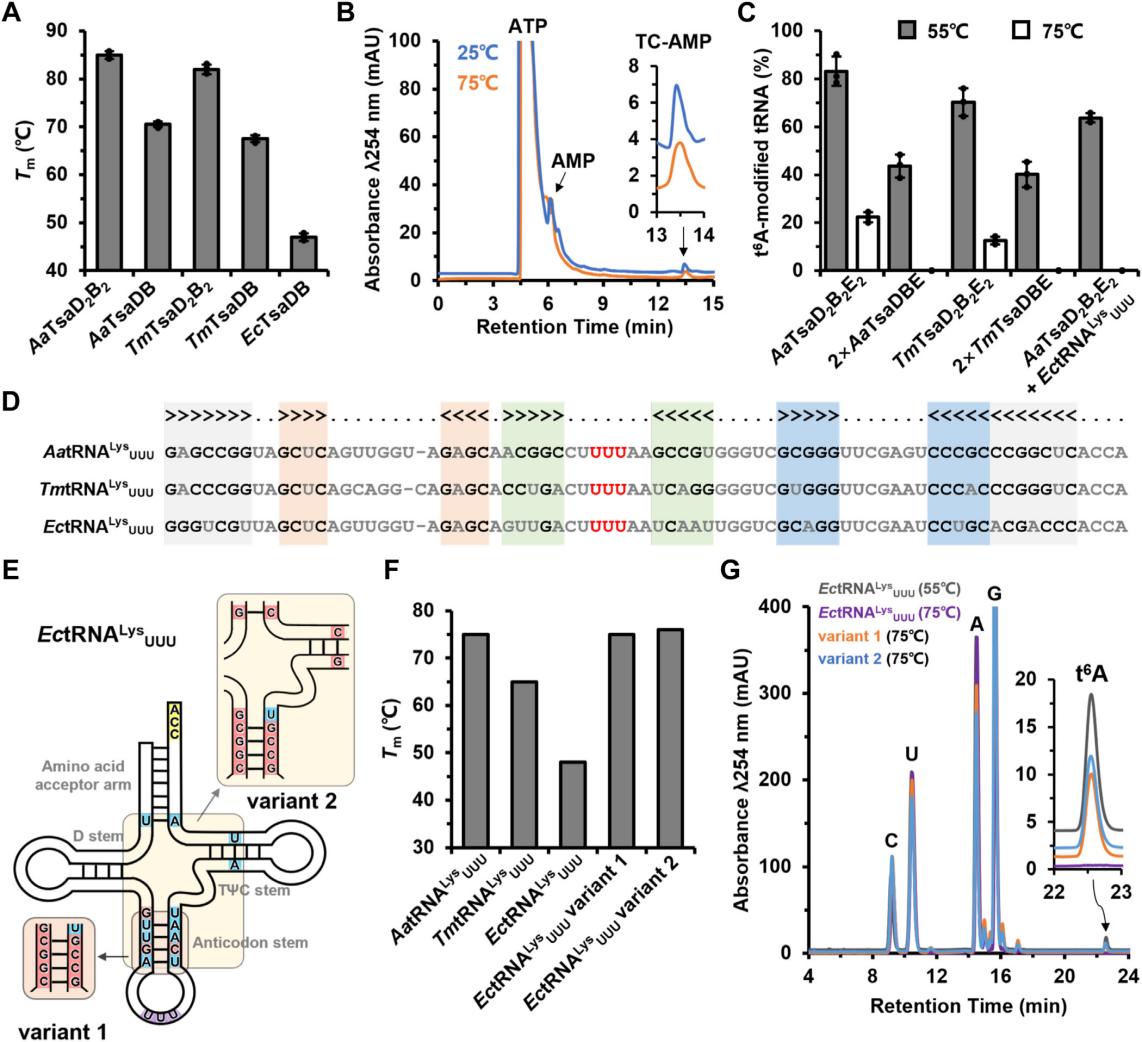
**Thermostability analysis of tRNA t**^**6**^**A biosynthetic systems from *Aquifex aeolicus*, *Thermotoga maritima* and *Escherichia coli*.***A*, melting temperature (*T*_m_) values of TsaD_2_B_2_ or TsaDB from *A*. *aeolicus*, *T*. *maritima*, or *E*. *coli* determined by thermal shift assay. Error bars represent standard deviations from triplicate measurements. *B*, LC–MS chromatograms of TC-AMP formation in assays that contained 5 μM *Aa*TsaC, 1 mM ATP, 4 mM *L*-threonine, and 10 mM NaHCO_3_ at 25 °C or 75 °C. *C*, quantification of tRNA t^6^A modification efficiency by 5 μM TsaD_2_B_2_E_2_ or 10 μM TsaDBE from *A*. *aeolicus* or *T*. *maritima* toward 60 μM cognate IVT tRNA^Lys^_UUU_ at 55 °C or 75 °C. For the assay with 60 μM *E.coli* tRNA^Lys^_UUU_, 5 μM *A.aeolicus* TsaD_2_B_2_E_2_ was used. Error bars represent standard deviations from triplicate measurements. *D*, sequence alignment of tRNA^Lys^_UUU_ from *A*. *aeolicus*, *T*. *maritima*, and *E*. *coli*. Amino acid acceptor arms, D–stems, anticodon stems, and TψC stems are shown in *gray*, *pink*, *green*, and *blue*, respectively. GC pairs and anticodons are highlighted in *black* and *red*, respectively. *E*, schematic representation of the GC base pairs mutations of *E*. *coli* tRNA^Lys^_UUU_ variant 1 and variant 2. *F*, *T*_m_ values of *A*. *aeolicus* tRNA^Lys^_UUU_, *T*. *maritima* tRNA^Lys^_UUU_, *E*. *coli* tRNA^Lys^_UUU_, *Ec*tRNA^Lys^_UUU_ variant 1 and variant 2, which were determined by circular dichroism spectra analysis. *G*, LC–MS chromatograms of tRNA t^6^A formation by *Aa*TsaC and *Aa*TsaD_2_B_2_E_2_ in assay using *E*. *coli* tRNA^Lys^_UUU_, variant 1 and variant 2 at 55 °C or 75 °C. IVT, *in vitro* transcribed; t^6^A, *N*^6^-threonylcarbamoyladenosine

*Aquificales* and *Thermotogales* grow optimally above 60 °C, and their enzymes are usually optimally active between 60 °C and 85 °C (54). Protein oligomerization is one of the molecular mechanisms that is responsible for the thermostability and optimal catalytic activity of thermophilic enzymes at higher temperatures (54). *A*. *aeolicus* and *T*. *maritima* TsaDBs share similar structural features and same catalytic mechanisms with *E*. *coli* TsaDB. We set out to analyze thermostability and biochemical properties of TsaDB and TsaD_2_B_2_ at different temperatures. Using thermal shift assay (Fig. S6, *A–E*), we first determined a melting temperature (*T*_m_) value of 70 °C, 67 °C, and 47 °C for *A*. *aeolicus* TsaDB, *T*. *maritima* TsaDB, and *E*. *coli* TsaDB, respectively (Fig. 5*A*). In contrast, the *T*_m_ value of *A*. *aeolicus* TsaD_2_B_2_ and *T*. *maritima* TsaD_2_B_2_ is 85 °C and 83 °C, respectively. Obviously, thermophilic TsaD_2_B_2_ exhibits better thermostability than thermophilic TsaDB and mesophilic TsaDB.

We first determined that *A*. *aeolicus* TsaC is equally active in generating TC-AMP in the presence of *L*-threonine, NaHCO_3_, and ATP at 25 °C and 75 °C (Fig. 5*B*). We tested the t^6^A-catalytic activities of TsaDBE–tRNA^Lys^_UUU_ and TsaD_2_B_2_E_2_–tRNA^Lys^_UUU_ in combination with *A*. *aeolicus* TsaC (for production of TC-AMP) at 55 °C and 75 °C (Figs. 5*C*, S3, *I* and *J*). Our quantification shows about 85% and 22% *A*. *aeolicus* tRNA^Lys^_UUU_ could be t^6^A-modifed by *A*. *aeolicus* TsaD_2_B_2_E_2_ at 55 °C and 75 °C, respectively. In contrast, only 43% *A*. *aeolicus* tRNA^Lys^_UUU_ could be t^6^A-modifed by two-fold *A*. *aeolicus* TsaDBE at 55 °C while almost no *A*. *aeolicus* tRNA^Lys^_UUU_ could be t^6^A-modifed by *A*. *aeolicus* TsaDBE at 75 °C. Likewise, *T*. *maritima* TsaD_2_B_2_E_2_ exhibits a higher t^6^A-catalytic activity toward *T*. *maritima* tRNA^Lys^_UUU_ at 55 °C (more than 70% t^6^A-modified tRNA) than that at 75 °C (less than 20% t^6^A-modified tRNA). The t^6^A-catalytic activity of *T*. *maritima* TsaD_2_B_2_E_2_ is almost doubled compared to that of two-fold *T*. *maritima* TsaDBE at 55 °C. *T*. *maritima* TsaDBE is inactive at 75 °C. Notably, both *A*. *aeolicus* TsaD_2_B_2_E_2_ and *T*. *maritima* TsaD_2_B_2_E_2_ exhibit higher t^6^A-catalytic activity at 55 °C (more than 70% t^6^A-modified tRNA) than those at 37 °C (less than 45% t^6^A-modified tRNA) (Figs. 2*E*, 5*C*), implying that thermophilic tRNA t^6^A-modifying enzymes are optimally active at 55 °C. Collectively, these results show that thermophilic TsaD_2_B_2_ tetramers are active at 75 °C while their TsaDB dimers are not active at above 75 °C. Our data demonstrate that dimerization of thermophilic TsaDBs promotes their thermostability and activity at higher temperatures.

### GC base pairs in anticodon stem of tRNA enhance thermostability

As *E*. *coli* TsaDB is likely inactive at 55 °C due to structural destabilization (Fig. 5*A*), we used *A*. *aeolicus* TsaD_2_B_2_E_2_ to test the t^6^A modification of *in vitro* transcribed (IVT) *E*. *coli* tRNA^Lys^_UUU_ at 55 and 75 °C. The results show that *E*. *coli* tRNA^Lys^_UUU_ is efficiently t^6^A-modified at 55 °C but not t^6^A-modified at 75 °C (Fig. 5*C*), suggesting that a t^6^A-productive structure of IVT *E*. *coli* tRNA^Lys^_UUU_ is lost at 75 °C. Nucleotide sequence alignment reveals that *A*. *aeolicus* tRNA^Lys^_UUU_ and *T*. *maritima* tRNA^Lys^_UUU_ contains 18 and 16 GC base pairs, respectively, whereas *E*. *coli* tRNA^Lys^_UUU_ contains only 13 GC base pairs. Variation in GC base pairs is particularly concentrated in anticodon stem of tRNA (Fig. 5, *D* and *E*). We first monitored the folding changes of these three IVT tRNAs^Lys^_UUU_ in course of increasing temperatures by circular dichroism (CD) spectra (Fig. S6, *F–H*), and determined that a *T*_m_ value of 75 °C, 65 °C, and 48 °C for *A*. *aeolicus* tRNA^Lys^_UUU_, *T*. *maritima* tRNA^Lys^_UUU_, and *E*. *coli* tRNA^Lys^_UUU_, respectively (Fig. 5*F*). To verify the role of GC base pairs in promoting thermostability of tRNA, we generated two variants of *E*. *coli* tRNA^Lys^_UUU_—tRNA^Lys^_UUU_^U28C/U29G/A31C/U39G/A41C/A42G^ (variant 1) and tRNA^Lys^_UUU_^U7G/U28C/U29G/A31C/U39G/A41C/A42G/A51G/U63C/A66C^ (variant 2). In *E*. *coli* tRNA^Lys^_UUU_ variant 1, three AU base pairs in anticodon stem were replaced with 3 GC base pairs; in *E*. *coli* tRNA^Lys^_UUU_ variant 2, two extra GC base pairs were introduced in acceptor arm and T-arm of *E*. *coli* tRNA^Lys^_UUU_ variant 1 (Fig. 5*E*). Remarkably, *T*_m_ values of *Ec*tRNA^Lys^_UUU_ variant 1 and *Ec*tRNA^Lys^_UUU_ variant 2 have increased to 75 °C and 76 °C, respectively (Fig. 5*F*), demonstrating that introduction of GC base pairs, particularly in anticodon stem, markedly enhances thermostability of *E*. *coli* tRNA^Lys^_UUU_. We further determined that about 35% tRNA^Lys^_UUU_ variant 1 and 40% tRNA^Lys^_UUU_ variant 2 could be t^6^A-modified by *A*. *aeolicus* TsaD_2_B_2_E_2_ at 75 °C (Fig. 5*G*). These data demonstrate that GC base pairs in anticodon stem play a pivotal role in folding and stabilizing tertiary structure of thermophilic tRNAs.

## Discussion

Here, we show that mesophilic and thermophilic TsaDB and TsaE proteins share remarkable structural and catalytic similarities (Fig. S2*B*). The conserved H–H–D motif of TsaD forms a metal-binding sphere that interacts directly with the phosphonate oxygen and carboxylate oxygens of ADP or TC-AMP (Fig. 1*D*) (27, 42, 43, 44). Mutation of H–H–D motif results in dead t^6^A-catalytic activities of TsaD (27, 42, 43) and Kae1/Qri7 proteins (18, 32, 36, 37). Our t^6^A-catalytic assay reveals that Fe^2+^, Mg^2+^, and Zn^2+^ could restore the dead t^6^A-catalytic activity of metal-depleted TsaD (Fig. 1*E*). Previous analyses showed that the TsaD domain of *P*. *salinus* TsaN relies on Mn^2+^ or Mg^2+^ (not Fe^2+^ and Zn^2+^) in catalyzing the threonylcarbamoylation modification of ATP and ADP (41); human mitochondrial OSGEPL1 is capable of using Fe^2+^, Mg^2+^, Zn^2+^, and Mn^2+^ for tRNA t^6^A modification (38, 39). This analysis demonstrates that not only the conserved H–H–D motif but the divalent metal ions play a pivotal role in the binding of TC-AMP and/or catalytic transfer of TC-moiety. It also implies that different metal ions with similar physiochemical properties (*i*.*e*. Fe^2+^, Mg^2+^, Zn^2+^, and Mn^2+^) may be exploited by TsaD/Kae1/Qri7 proteins under different cellular contexts (61). In t^6^A-catalytic center of *A*. *aeolicus* TsaD, we identified an oxidized state of Cys10 (CSO10), which is in range of van der Waals force contact of the TC-moiety of TC-AMP (about 5 Å). We rerefined the crystal structure of *E*. *coli* TsaD–TsaB in complex with BK951 and also found well-defined density for CSO10 (data not shown) (42). CSO10 is part of an ETSCD motif conserved in bacterial TsaD proteins and mitochondrial Qri7/OSGEPL1 proteins (Fig. S2*C*). Mutation of Cys10 significantly compromises the t^6^A-catalytic activity of *A*. *aeolicus* TsaD (Fig. 1*E*) and *E*. *coli* TsaD (42). Interestingly, different double substitutions of Ser9 and Cys10 of *E*. *coli* TsaD lead to very different extent to which the t^6^A-catalytic activity is affected (42). *E*. *coli* TsaC is able to use hydroxynorvaline (hnV) and *L*-serine to generate intermediates hnC-AMP and SC-AMP, respectively. hnC-AMP can be used by *E*. *coli* TsaD to hn^6^A-modify tRNAs; SC-AMP cannot be used by TsaD to SC-modify tRNAs (42, 62). The inactivity of TsaD on SC-AMP suggests that Cys10 or the ETSCD motif participates in stabilizing the threonyl-moiety and in assisting the nucleophilic attack on carbonyl-group by tRNA A37 *via* a metal ion (42, 45, 56, 61, 62).

It now seems a universal mechanism that bacterial TsaDB possesses a basal t^6^A-catalytic activity that is further necessarily stimulated by TsaE (Fig. 2*E*) (43). Stimulation of t^6^A-catalytic activity of TsaDB by TsaE is coupled with ATP to ADP hydrolysis by TsaE, which in turn regulates the binding of TsaE to TsaDB (Fig. 1*A*). Structure and sequence alignments exhibit that the interacting residues at the interfaces of *T*. *maritima* TsaDB and TsaE are less conserved in TsaDB and TsaE from *A*. *aeolicus* and *E*. *coli* (Table S2), which explains the ATP-dependence of interaction between TsaDB and TsaE from *A*. *aeolicus* (Fig. S4*A*) and *E*. *coli* (27). Both the ATPase activity (Fig. 4*A*) and t^6^A-catalytic activity of *E*. *coli* TsaDBE (Fig. 2*E*) are significantly higher than TsaD_2_B_2_E_2_ from *A*. *aeolicus* and *T*. *maritima*, manifesting a correlation between a quicker ATP to ADP hydrolysis by TsaE and more efficient t^6^A-catalysis by TsaD. Our kinetic analysis of ATP hydrolysis reveals a coupled communication between t^6^A-catalytic activation of TsaDB and ATP hydrolysis-regulated binding of TsaE to TsaDB. TsaDB promotes the ATP-hydrolysis rate of TsaE but decreases the apparent binding affinity of ATP to TsaE (Table 2). It was determined that *E*. *coli* TsaD has a higher binding affinity toward ATP (*K*_d_ = 0.7 μM) than TC-AMP (*K*_d_ = 3.4 μM) (27, 42). The cellular level of ATP is much higher than the low-yielding and chemically unstable TC-AMP. Thus, one key issue is the exchange of bound adenine nucleotides (ATP, ADP, and AMP) by TC-AMP in the catalytic site of TsaD. Models of TsaDB–tRNA and KEOPS–tRNA reveal extensive steric clashes between anticodon loop and the N-terminal subdomain of TsaD/Kae1 (43, 49). Binding of tRNA A37 in the catalytic site of TsaD/Kae1/Qri7 proteins likely involves mutual conformational changes of both tRNA and TsaD/Kae1/Qri7 proteins. Crystal structures of *T*. *maritima* TsaDBE and *A*. *aeolicus* TsaDB reveal that binding of TsaE to TsaDB induces conformational changes of α6, α7, α8, and helical hairpin α1–α2 of TsaD (Fig. S2*F*). Removal of α1 of *A*. *aeolicus* TsaD sustains the formation of *A*. *aeolicus* TsaDBE complex (Fig. S4*E*) and promotes the binding of tRNA to *A*. *aeolicus* TsaDB (Fig. S4*K*), but leads to almost dead t^6^A-catalytic activity (Fig. 3*J*). It suggests that TsaE possibly regulates the binding of TC-AMP and release of AMP after t^6^A-catalysis (Fig. 1*A*) *via* modulating the structural conformation of α1, α2, and α7 of TsaD (Fig. S2*F*). Contact of α6 and α8 of TsaD and switch II of TsaE stimulates hydrolysis of ATP to ADP by TsaE, which leads to dissociation of ADP-bound TsaE from TsaDB and creates a specific configuration for subsequent binding of TC-AMP.

Few studies have reported the binding affinities for tRNAs and TsaDB/KEOPS (9). Luthra *et al*. determined a *K*_d_ value of 1.2 μM for *T*. *maritima* TsaD_2_B_2_ and *E*. *coli* tRNA^Thr^_CGU_ by EMSA (28); Beenstock *et al*. determined a *K*_d_ value of 263 nM for *Methanocaldococcus jannaschii* KEOPS and tRNA by fluorescence polarization assay (49); Zheng *et al*. determined a *K*_d_ value of 18 μM for *Aquifex thaliana* KEOPS and tRNA^Arg^_CCU_ by MST (36). Here, we show strong interactions between *T*. *maritima* TsaD_2_B_2_ and tRNA^Lys^_UUU_ (*K*_d_ ≈ 1.20 μM, Fig. 4, *J* and *M*), and between *A*. *aeolicus* TsaD_2_B^mut^_2_ and tRNA^Lys^_UUU_ (*K*_d_ ≈ 1.16 μM, Fig. 4, *L* and *M*) by EMSA. While a stable complex of *A*. *aeolicus* TsaD_2_B_2_E_2_ is formed (Figs. 2*C*, 3*F*), an *A*. *aeolicus* TsaD_2_B_2_–tRNA complex was never confirmed by SEC under different conditions (data not shown). Models of TsaDBE–tRNA show that the C-terminal tails of TsaBs contact the bound TsaE and tRNAs (Figs. 2*B*, 3*A*). Deletion of C tail of TsaB does not affect the binding of TsaE to TsaDB (27) but interferes the interactions between TsaDB and tRNA (43). Our “gain-of-function” mutant—*A*. *aeolicus* TsaD_2_B^mut^_2_—exhibits markedly higher binding affinity toward tRNA^Lys^_UUU_ (Figs. 4*L*, 3*I*) and t^6^A-catalytic activity (Fig. 3*J*), documenting a pivotal role of the C-terminal tail of TsaB in binding tRNA. According to our docked model of *A*. *aeolicus* TsaD_2_B_2_–TsaE–tRNA (Fig. 3*A*) and SAXS model of *T*. *maritima* TsaD_2_B_2_–tRNA, two molecules of tRNA could be simultaneously bound to two TsaDB protomers of TsaD_2_B_2_. However, Luthra *et al*. determined one molecule of tRNA is bound to TsaD_2_B_2_ or TsaD_2_B_2_E (28). Our results show that TsaD_2_B_2_ tetramers possess higher binding affinities toward tRNAs than TsaDB dimers (Fig. 2*D*) and exhibit higher t^6^A-catalytic activities than two-fold TsaDB dimers (Fig. 2*E*). In addition, *E*. *coli* TsaDB dimer is more active than equimolar TsaD_2_B_2_ tetramers. It can be drawn from these data that dimerization of TsaDB dimer induces conformational changes of TsaD_2_B_2_ that favors binding of only one molecule of tRNA. Such a mechanism is also adopted by *S*. *cerevisiae* Qri7 homodimer, to which one molecule of tRNA is bound to one protomer with support of the other protomer (37). On the other hand, the oligomerization of TsaDB creates an enlarged hydrophobic area and markedly promotes thermostability of TsaD_2_B_2_ (Fig. 5*A*), which is needed property for thermophilic TsaDBs to function at above 60 °C (54). Such an oligomerization-based mechanism is also seemingly adopted by archaean tRNA t^6^A-modifying enzyme–KEOPS complex, which dimerizes *via* Pcc1 dimerization (30, 32, 63).

We show that the *T*_m_ values of *A*. *aeolicus* and *T*. *maritima* tRNA^Lys^_UUU_s are significantly higher than that of *E*. *coli* tRNA^Lys^_UUU_ (Fig. 5*F*). Introduction of 3 GC base pairs in anticodon stem of *E*. *coli* tRNA^Lys^_UUU_ confers strikingly better thermostability (*T*_m_ value increases by 26 °C) (Fig. 5*F*) and restores the t^6^A-catalysis of *A*. *aeolicus* TsaD_2_B_2_E_2_–*E*. *coli* tRNA^Lys^_UUU_ variant 1 (Fig. 5*G*). Of note, the t^6^A-modification frequencies of thermophilic TsaD_2_B_2_E_2_–tRNAs are higher at 55 °C than 37 °C but decreases substantially at 75 °C (Figs. 2*E*, 5*C*). We assume that such differences are mainly due to compromised thermostability of IVT tRNAs^Lys^_UUU_ due to the absence of other core posttranscriptional modifications that help stabilize the folding and tertiary structures of tRNAs (64, 65). *Thermotogales* and *Aquificales* are the deepest branches in the bacterial genealogy, the conserved tRNA t^6^A biosynthetic systems are remarkable targets for investigation of the mechanisms underscoring the thermostability of thermophilic proteins. Our structures and biochemical data also represent an obvious interest in evolutionary studies of molecular structures of ancient proteins. The catalytic mechanisms of tRNA t^6^A by bacterial TsaDBE complex remain to be completely understood.

## Experimental procedures

### Bioinformatics

We performed sequence alignment and calculated the sequence identities using Clustal Omega (66). *Aa*TsaD (Uniprot: O66986) was aligned to *Tm*TsaD (Uniprot: Q9WXZ2) and *Ec*TsaD (Uniprot: P05852); *Aa*TsaB (Uniprot: O66494) was aligned to *Tm*TsaB (Uniprot: Q9WZX7) and *Ec*TsaB (Uniprot: P76256); *Aa*TsaE (Uniprot: O67011) was aligned to *Tm*TsaE (Uniprot: Q9X1W7) and *Ec*TsaE (Uniprot: P0AF67). Structural alignment, calculation of RMSD values and visualization were carried out using PyMOL (https://pymol.org) (Schrödinger, LLC). Pairwise interacting residues at the interfaces of TsaD–TsaB–TsaE were calculated by PDBePISA (67). AlphaFold prediction structures of *Aa*TsaE (AF ID: O67011) and *Ec*TsaE (AF ID: P0AF67) were retrieved from the AlphaFold Protein Structure Database (https://alphafold.ebi.ac.uk) (59). *Aa*, *A*. *aeolicus*; *Tm*, *T*. *maritima*; *Ec*, *E*. *coli*.

### Cloning, mutagenesis, expression, and purification

The polycistronic plasmid pET21a–BC10_DZop that simultaneously expresses *A*. *aeolicus* TsaD and TsaB with a six-histidine (6His) tag added at the C terminus of TsaB, and the plasmid pET24d–BC11_Eop that expresses *A*. *aeolicus* TsaE were provided by Herman van Tilbeurgh’s laboratory. The plasmid pET21a–*Aa*–TsaC that expresses *A*. *aeolicus* TsaC with a 6His tag at the C terminus was constructed using a chemically synthesized DNA (Tsingke) and a pET21a vector using restriction sites of *Nde*I and *Xho*I. Plasmids pET9a–ygjD, pET9a–yeaZ, and pET28a–yjeE that express *E*. *coli* TsaD, *E*. *coli* TsaB, and *E*. *coli* TsaE, respectively, were generated by Patrick Forterre’s laboratory (68) and provided by Herman van Tilbeurgh’s laboratory (27). The plasmids pET21a–BC14 and pET21a–BC15 that express *T*. *maritima* TsaD–TsaB and *T*. *maritima* TsaE, respectively, were provided by Herman van Tilbeurgh’s laboratory (28). PCR-based site directed mutagenesis was carried out by In-Fusion HD Cloning Plus according to the manufacturer’s protocols using primers listed in Table S3. *A*. *aeolicus* TsaDB heterodimer was generated by mutating residues (E21A, K22A, V23A, T24A, F25A, L26A, H27A, Y28A, L29A, K30A, K98A, D159A, I160A, and Y166A) of TsaB while *T*. *maritima* TsaDB heterodimer was generated by mutating residues (E20A, D21A, L22A, E24A, S26A, Y27A, T28A, E30A, and K31A) of TsaB.

Proteins were expressed in *E*. *coli* BL21 (DE3) and *E*. *coli* Rosetta pLySs cells that were transformed with corresponding plasmids following protocols in previous studies (27). In general, cells harboring plasmids were grown at 37 °C in 2YT medium supplemented with corresponding antibiotics (100 μg/ml ampicillin or 50 μg/ml kanamycin in their final concentration) and induced with 0.5 mM IPTG when the *A*_600_ reaches 0.6 to 0.8. Cells were grown for 3 h at 37 °C and harvested by centrifugation. Cell pellets were resuspended in lysis buffer (20 mM Tris–HCl pH 7.5, 300 mM NaCl, and 5 mM β-mercaptoethanol) and lysed by sonication on ice. Lysates were clarified by centrifugation at 8 °C and the supernatant was applied to affinity chromatography using a Ni-NTA resins for initial purification. Imidazole-eluted proteins were analyzed by SDS–PAGE and applied to SEC using a HiLoad 16/600 Superdex 200 prep grade column (GE HealthCare). Protein fractions were analyzed by SDS–PAGE and concentrated by centrifugal filtration (Millipore). Final protein concentration was determined by BioPhotometer D30 (Eppendorf). Purified proteins were used immediately or flash-frozen in liquid nitrogen and stored at −80 °C.

For the assembly between *A*. *aeolicus* TsaDB dimer and TsaE, analytical SEC experiments were performed on a calibrated Superdex 75 10/300 GL column (GE HealthCare). Briefly, 500 μl sample containing 400 μg TsaDB dimer and 450 μg TsaE in buffer A containing 20 mM Tris–HCl pH 7.5, 200 mM NaCl, 5 mM β-mercaptoethanol, 1 mM ATP, and 2 mM MgCl_2_ were loaded on the column and run using buffer A, at a flow rate of 0.5 ml/min, with detection at 280 nm. The formation of *Aa*TsaDBE complex was analyzed by SDS–PAGE.

### Crystallization, X–ray data collection, and structural determination

Crystallization of *A*. *aeolicus* TsaD–TsaB was initially carried out with Gryphon Crystallization Robot (Art Robbins). Final crystals of TsaD–TsaB were grown in sitting drops at 18 °C by mixing 1 μl of *A*. *aeolicus* TsaD_2_B_2_ protein solution (9 mg/ml in 20 mM Tris–HCl and 300 mM NaCl) and 1 μl of reservoir solution that contained 0.1 M MES pH 6.5, 30% (v/v) PEG300. The crystals of TsaD–TsaB in complex with ADP were grown in the same mixture that was supplemented with 5 mM ATP and 5 mM MgCl_2_. Single crystal was cryo-protected in 30% glycerol and directly mounted in a micro loop (MiTeGen) and flash–frozen in liquid nitrogen for data collection. Diffraction data were collected at 150 K with an in-house X-ray diffraction system (RIGAKU FR-X). Crystallographic datasets were processed with CrysAlisPro software (https://rigaku.com/products/crystallography/x-ray-diffraction/crysalispro) and scaled using AIMLESS in the CCP4 suite (69). The structure of *A*. *aeolicus* TsaD and TsaB was solved by molecular replacement using MOLREP v11.9.02 in CCP4 suite using *E*. *coli* TsaD (PDB: 4YDU) and AlphaFold model of *A*. *aeolicus* TsaB (AF ID: A0A7C5Q8I2) as search templates (59). Model building and refinement was performed using COOT (https://www2.mrc-lmb.cam.ac.uk/personal/pemsley/coot/) (70) and REFMAC (https://www.ccp4.ac.uk/html/refmac5.html) (71), respectively. The final model was validated by the PDB validation server. Structural representations were generated using PyMol (Schrödinger, Inc).

### tRNA transcription and purification

For *in vitro* transcription of tRNAs, the template DNAs for tRNAs were amplified by PCR using primers, with the 5′ terminus containing an in-frame T7 recognition sequence (Table S3). Transcription was carried out overnight at 37 °C in a 1000 μl reaction mixture composed of 40 mM Tris–HCl pH 8.0, 4 mM NTPs, 15 mM GMP, 5 mM DTT, 2 mM spermidine, 0.5% Triton X-100, 30 mM MgCl_2_, 120 ng/μl DNA templates, 5 μM T7 RNA polymerase, and 5 μM pyrophosphatase. *In vitro* transcripts were separated *via* 12% Urea–PAGE and visualized using a hand-held UV light. The shadowed bands in the gel were cut out and placed in 30 ml extraction buffer containing 0.5 M NaAc pH 5.2, 1 mM EDTA and 0.5% SDS. tRNA transcripts were extracted from the gel by crushing and soaking at 37 °C overnight, followed by precipitation with addition of 90 ml 100% ethanol and cooling at −80 °C for 8 h. tRNA precipitates was collected by centrifugation at 13,000 rpm for 15 min at 8 °C and resuspended in buffer containing 20 mM Tris–HCl pH 8.0, 100 mM KCl, and 5 mM MgCl_2_. The refolding of tRNA was performed by heating up to 95 °C for 5 min and gradually cooling down in water bath to room temperature. The concentration of tRNA was measured by NanoDrop 2000 (Thermo Fisher Scientific), and the correct folding was confirmed with CD spectroscopy.

### Enzymatic synthesis of TC-AMP

TC-AMP was generated in a 10 μl reaction mixture containing 5 μM *A.* *aeolicus* TsaC, 1 mM ATP, 4 mM *L*-threonine, and 10 mM NaHCO_3_ in assay buffer (20 mM Tris−HCl pH 8.0, 300 mM NaCl, 1 mM MgCl_2_, and 1 mM DTT) at 25 °C. To test the thermostability of *A*. *aeolicus* TsaC, formation of TC-AMP at 55 °C and 75 °C was also analyzed. TC-AMP compound was prepared by isolation of TC-AMP solution and freeze-drying following our previous protocol (41).

### Enzymatic synthesis of tRNA t^6^A

Briefly, 5 μM TsaC, 5 μM TsaD_2_B_2_ (or 10 μM TsaDB), 10 μM TsaE, 60 μM IVT tRNA^Lys^_UUU_ from *A*. *aeolicus* or *T*. *maritima*, 5 mM ATP, 10 mM *L*-threonine, and 15 mM NaHCO_3_ were incubated at 37 °C for 90 min to generate tRNA t^6^A. For *E*. *coli* proteins, 5 μM TsaDB and 5 μM TsaE were applied. For analysis of the metal identity, metal-free tRNA t^6^A assay was performed with EDTA-dialyzed *A*. *aeolicus* TsaD_2_B_2_, SEC-purified tRNA^Lys^_UUU_ and TC-AMP compound. For the thermostability analysis, formation of tRNA t^6^A were carried in 200 μl Eppendorf tubes at 55 °C or 75 °C on a metal heating block. In order to precisely quantify the t^6^A formation, t^6^A-tRNAs out of the assays were further purified using 12% Urea−PAGE, followed by nucleoside digestion using 0.1 U/μl nuclease P1 (Sigma-Aldrich) and 0.05 U/μl alkaline phosphatase (Sigma-Aldrich).

### Liquid chromatography–mass spectrometry analysis

Briefly, 10 μl mixture of digested nucleosides was applied to LC–MS using an Agilent 1260 Infinity equipped with an Agilent 6125B mass spectrometer. The nucleosides of A, U, C, G, and t^6^A was chromatographed using a C18 column (5 μm, 250 × 4.6 mm, Ecosil) and eluted with 1‰ trifluoroacetic acid (solvent A) and acetonitrile containing 1‰ trifluoroacetic acid (solvent B) at a flow rate of 0.8 ml/min with a multistep gradient: 2% solvent B for 5 min, 2 to 20% solvent B for 15 min, 20 to 98% solvent B for 7 min, and then initialized with 98% solvent B for 2 min. The retention time and mass-to-charge ratio of these nucleosides were collected using OpenLab CDS ChemStation Edition Online (Agilent). The UV peak areas were integrated, summed, and converted to the number of nucleosides using standard curves of these nucleosides–A, U, C, G (Sigma-Aldrich), and t^6^A (41). The t^6^A-catalytic activity was averaged by ratios of the number of t^6^A to A, U, C, or G, respectively, and normalized to calculated ratios according to sequence of IVT tRNA.

### Native gel shift assay

Protein–protein and protein–tRNA interactions were analyzed on native agarose-glycine gel in prechilled running buffer that contained 20 mM Tris–HCl and 50 mM glycine pH 8.5. Subsequently, 15 μg TsaD_2_B_2_ or variants and two-fold TsaE (in molar concentration) were mixed in loading buffer containing 20 mM Tris–HCl pH 7.5, 300 mM NaCl, and 25% (v/v) glycerol, and loaded onto a 1.6% agarose gel that was run at 100 V for 80 min. For analysis of the ATP-dependent interaction between TsaD_2_B_2_ or variants and TsaE, the gel and protein samples were supplemented with 1 mM ATP and 2 mM MgCl_2_. For analysis of the interaction between TsaD_2_B_2_ or variants and tRNA, 5′ 6-FAM (6-carboxyfluorescein)-labeled *Aa*tRNA^Lys^_UUU_ (5′ 6-FAM-*Aa*tRNA^Lys^_UUU_) and *Tm*tRNA^Lys^_UUU_ (5′ 6-FAM-*Tm*tRNA^Lys^_UUU_) were chemically synthesized (Tsingke) and were refolded before use. Subsequently, 1 μM 5′ 6-FAM-*Aa*tRNA^Lys^_UUU_ or 5′ 6-FAM-*Tm*tRNA^Lys^_UUU_ and 0 to 100 μM TsaD_2_B_2_ proteins or variants were incubated in a 10 μl mixture. For the competition assay, 1 μM 5′ 6-FAM-*Aa*tRNA^Lys^_UUU_, 5 μM TsaD_2_B^mut^_2_ and 0 to 20 μM TsaE were incubated with 1 mM ATP and 2 mM MgCl_2_. The mixture was loaded onto a 2% agarose gel and run at 100 V for 45 min. The presence of 5′ 6-FAM-tRNA^Lys^_UUU_ was visualized at a wavelength of 488 nm by Pharos FX (Bio-Rad), and protein bands were stained using Coomassie brilliant blue. Band densities were measured using ImageJ 1.54 g (https://imagej.net/ij), and the density data were fit using the nonlinear least squares method in GraphPad Prism 8.0.2 (https://www.graphpad.com).

### Microscale thermophoresis

MST experiments were performed on a Monolith NT.115 instrument (NanoTemper Technologies) in interaction buffer containing 20 mM PBS pH 7.5, 300 mM NaCl and 0.05% (v/v) Tween 20. 10 nM 5′ 6-FAM-labelled *Aa*tRNA^Lys^_UUU_ or *Tm*tRNA^Lys^_UUU_ was incubated with *Aa*TsaD_2_B_2_, *Tm*TsaD_2_B_2_, *Ec*TsaDB or variants at increasing concentrations (0.1953125, 0.390625, 0.78125, 1.5625, 3.125, 6.25, 12.5, 25, 50, and 100 μM). Measurements were performed at 25 °C in capillaries (MO-K022, NanoTemper Technologies) using 40% Excitation Power and medium MST Power. Binding data were analyzed using MO.affinity analysis software (https://support.nanotempertech.com) (NanoTemper Technologies). MST traces within 1 s before infrared excitation and within 4 to 5 s after infrared excitation were applied to generate the binding profiles using a quadratic equation binding *K*_d_ model. All experiments were reproduced for at least three times using proteins from different batches. Figures were created using GraphPad Prism 8.0.2.

### Isothermal titration calorimetry

ITC measurements were performed with MicroCal PEAQ-ITC. 10 μM *A.* *aeolicus* TsaD_2_B_2_, TsaD^Δ34–38^_2_B_2_ or TsaD_2_B^mut^_2_ and 240 μM TsaE were equilibrated in 20 mM Tris–HCl pH 7.5, 200 mM NaCl, 1 mM AMPPNP, and 2 mM MgCl_2_. For each measurement, TsaD_2_B_2_ or mutant was titrated with a total of 19 injections of 2 μl TsaE at intervals of 150 s under continuous stirring at 35 °C. A single-site binding model was fit to the data by a nonlinear regression analysis. The stoichiometry (N), binding affinity (*K*_d_), and enthalpy (ΔH) were analyzed using MicroCal PEAQ-ITC Analysis Software (https://www.malvernpanalytical.com/en/support/product-support/software/microcal-peaq-itc-analysis-software-v141).

### ATPase activity assay

The hydrolysis of ATP to ADP was quantified using a NADH–coupled assay, following the protocols previously reported (27). In the assay, the consumption of NADH is converted to the generation of ADP using a standard curve titrated with ADP (Sigma-Aldrich). Subsequently, 10 μM TsaE with the addition of 5 μM TsaD_2_B_2_ and 10 μM *Aa*tRNA^Lys^_UUU_ were added to 200 μl reaction mixture containing 100 mM Tris–HCl pH 7.5, 100 mM KCl, 10 mM MgCl_2_, 0.5 mM NADH (Sigma-Aldrich), 4 mM phospho(enol)pyruvic acid (Sigma-Aldrich) and 1 mM pyruvate kinase/lactate dehydrogenase (PK/LD) (Sigma-Aldrich) and varying concentrations of ATP (Sigma-Aldrich). Assays were performed in a 96-well plate at 37 °C. The consumption of NADH was monitored by measuring the absorbance at 340 nm at an interval of 30 s for 4 h by a spectrophotometer (Multiskan GO). ATP to ADP hydrolysis rate against ATP concentration was fitted and the steady-state kinetic parameters were calculated using Michaelis–Menten equation. All the experiments were performed in triplicates.

### Protein thermostability assay

The thermostability of proteins was evaluated using a fluorescence thermal shift assay (72). Subsequently, 5 μM proteins were added to 40 μl assay buffer (20 mM Tris–HCl pH 7.5 and 300 mM NaCl) supplemented with a final concentration 6xSYPRO Orange dye (Invitrogen) in a 96-well format PCR plate. The plate was heated in a StepOnePlus Real-Time PCR System (Thermo Fisher Scientific) from 25 °C to 99 °C at a rate of 0.5°C/min. The fluorescence was monitored using λ_ex_ = 490 nm and λ_em_ = 575 nm. The melting temperature (*T*_m_) values were calculated by plotting the first derivative of the fluorescence emission as a function of temperature (–dF/dT) *via* StepOne Software v2.3 (https://stepone-software.software.informer.com/2.3/). All the experiments were performed in biological triplicates.

### CD spectroscopy

CD was used to monitor the change in the secondary structure of the tRNA on thermal denaturation (73). Briefly, 0.3 mg/ml tRNAs in 300 μl RNAase-free water was heated from 20 °C to 95 °C at a rate of 5°C/min and the CD spectra from 190 nm to 320 nm were recorded by J-1500 CD Spectrometer (Jasco Corporation). *T*_m_ values were calculated by plotting the first derivative of the molar ellipticity (θ) values as a function of temperature (–dθ/dT) at 265 nm wavelength.

## Data availability

The coordinates of TsaD–TsaB–Fe^2+^ structure and TsaD–TsaB–ADP–Fe^2+^ structure were deposited in Protein Data Bank (www.rcsb.org) under accessions of 8IEY and 8IFX, respectively.

## Supporting information

This article contains supporting information.

## Conflict of interest

The authors declare that they have no conflicts of interest with the contents of this article.

## Supporting information

JBC-D-24-01912-SI-R2
