# Supplementary material for: Structure–function analysis of tRNA t6A-catalysis, assembly, and thermostability of Aquifex aeolicus TsaD2B2 tetramer in complex with TsaE
Source: J Biol Chem. 2024 Nov 5;300(12):107962. doi: 10.1016/j.jbc.2024.107962 (PMC11648230; doi:10.1016/j.jbc.2024.107962)
Supplement: JBC-D-24-01912-SI-R2 [file mmc1.docx]

**SUPPORTING INFORMATION**

**Structure–function analysis of tRNA t^6^A-catalysis, assembly and thermostability of *Aquifex aeolicus* TsaD_2_B_2_ tetramer in complex with TsaE**

Shuze Lu, Mengqi Jin, Zhijiang Yu and Wenhua Zhang^*^

School of Life Sciences, Key Laboratory of Cell Activities and Stress Adaptation of the Ministry of Education, Lanzhou University, Lanzhou 730000, China

^*^ Corresponding author, E-mail: zwh@lzu.edu.cn; Tel. & Fax: +86-931-8914381

**Running title**: Structure and functional properties of *A. aeolicus* TsaD_2_B_2_ tetramer

**Keywords**: *Aquifex aeolicus,* tRNA t^6^A, enzymatic reconstitution, crystal structure, TsaD_2_B_2_ tetramer, TsaD–TsaB–TsaE–tRNA assembly, ATP hydrolysis, oligomerization, GC base pairs content, thermostability

**Supplementary Table 1.** Data processing and refinement statistics for X-ray crystallography.


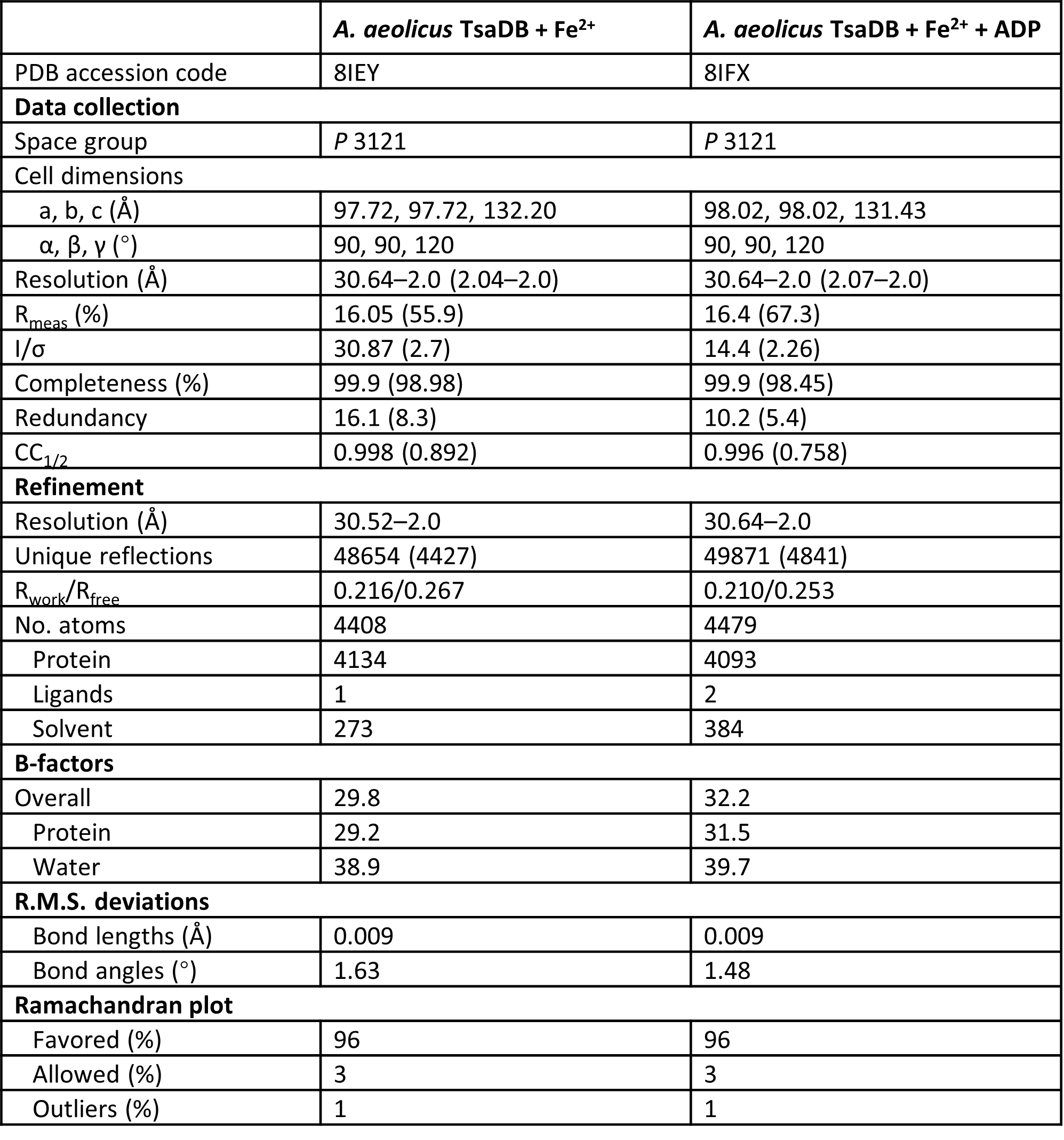


**Supplementary Table 2.** Pairwise interacting residues at the interfaces of TsaD–TsaB–TsaE complexes analyzed by PDBePISA. * denotes the conserved pairs of interacting resides based on sequence alignment.


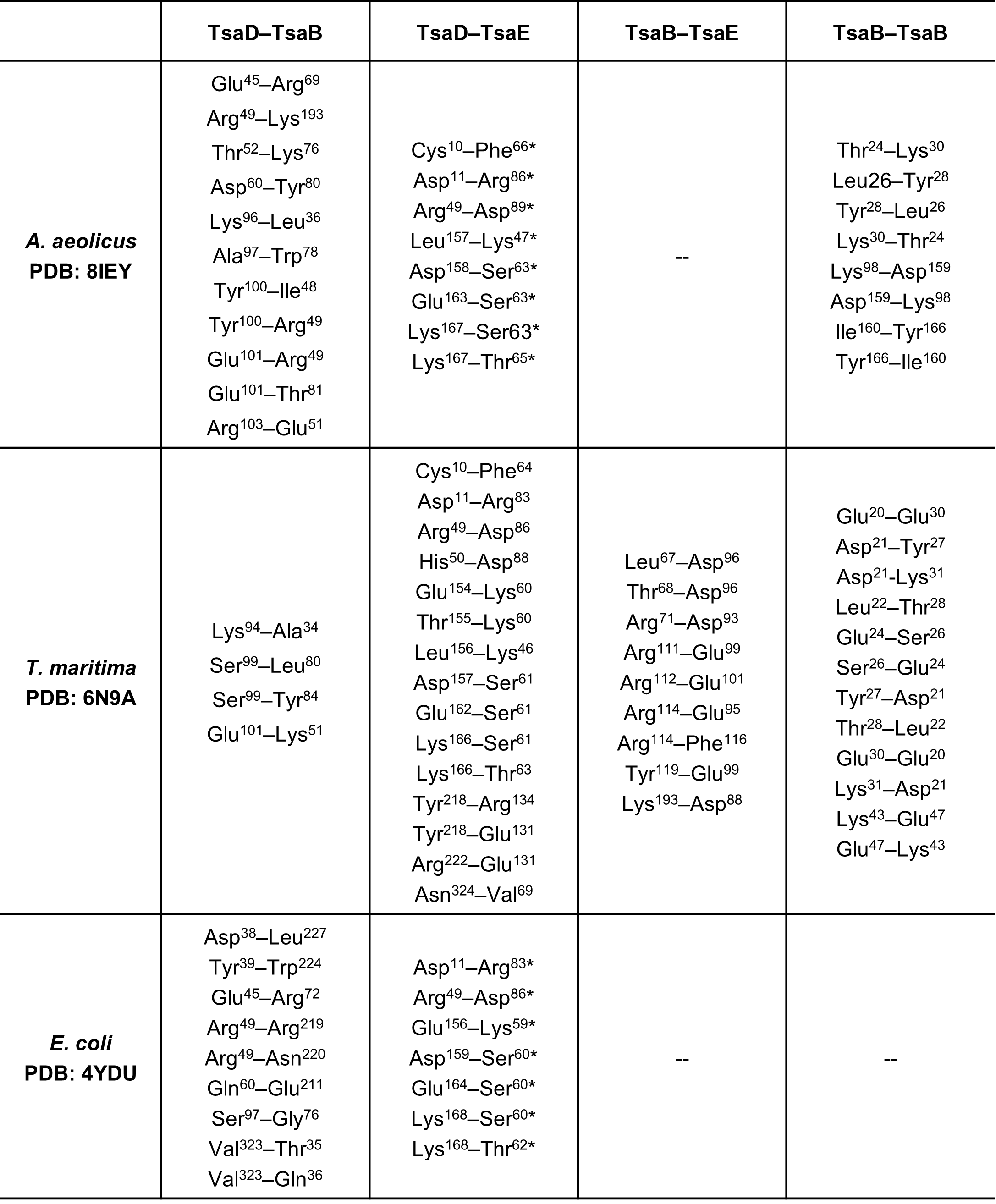


**Supplementary Table 3.** List of primers used for protein mutagenesis and DNA templates for tRNA transcription. *Aa*, *A. aeolicus*; *Tm*, *T. maritima*; *Ec*, *E. coli*.


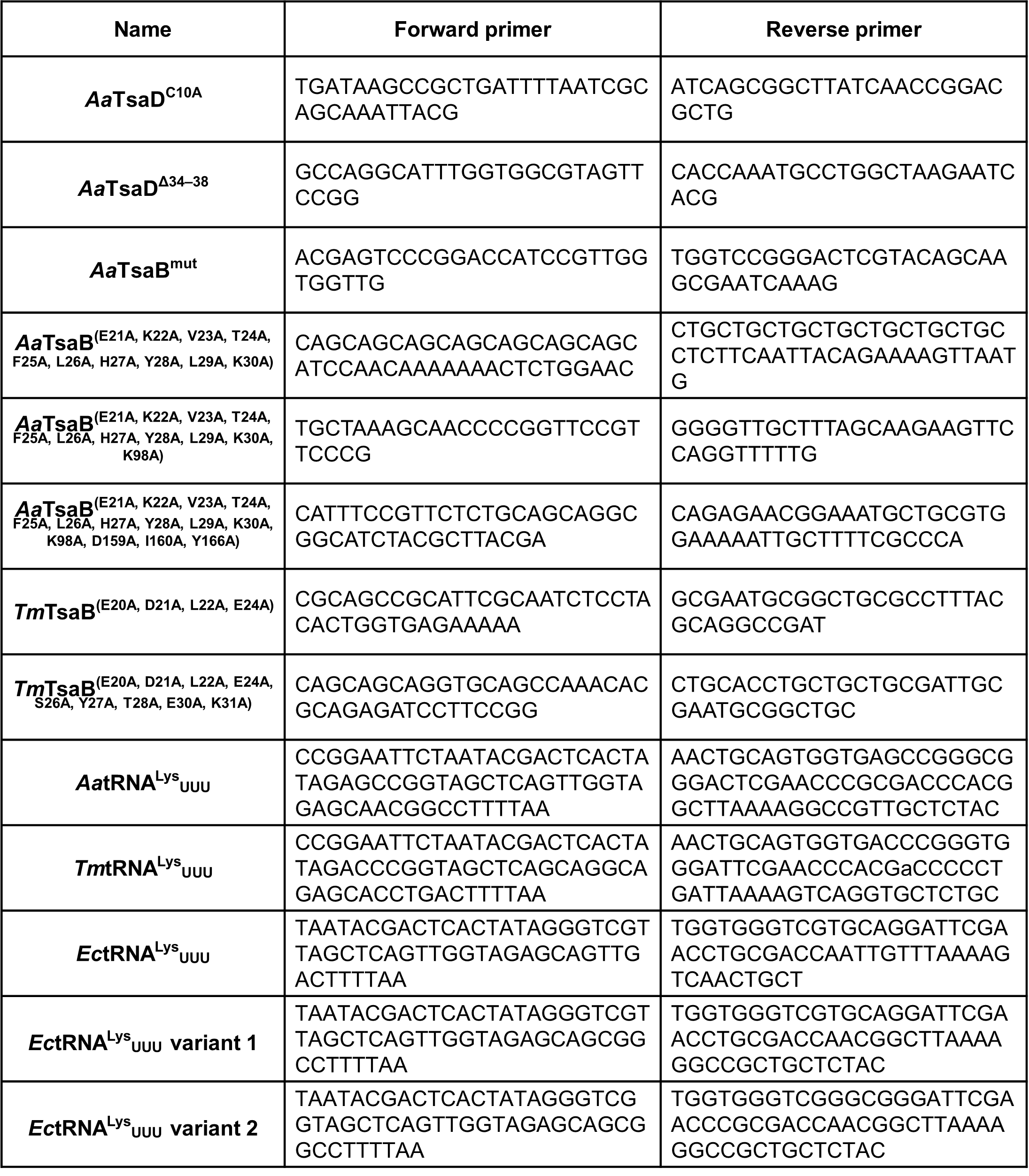


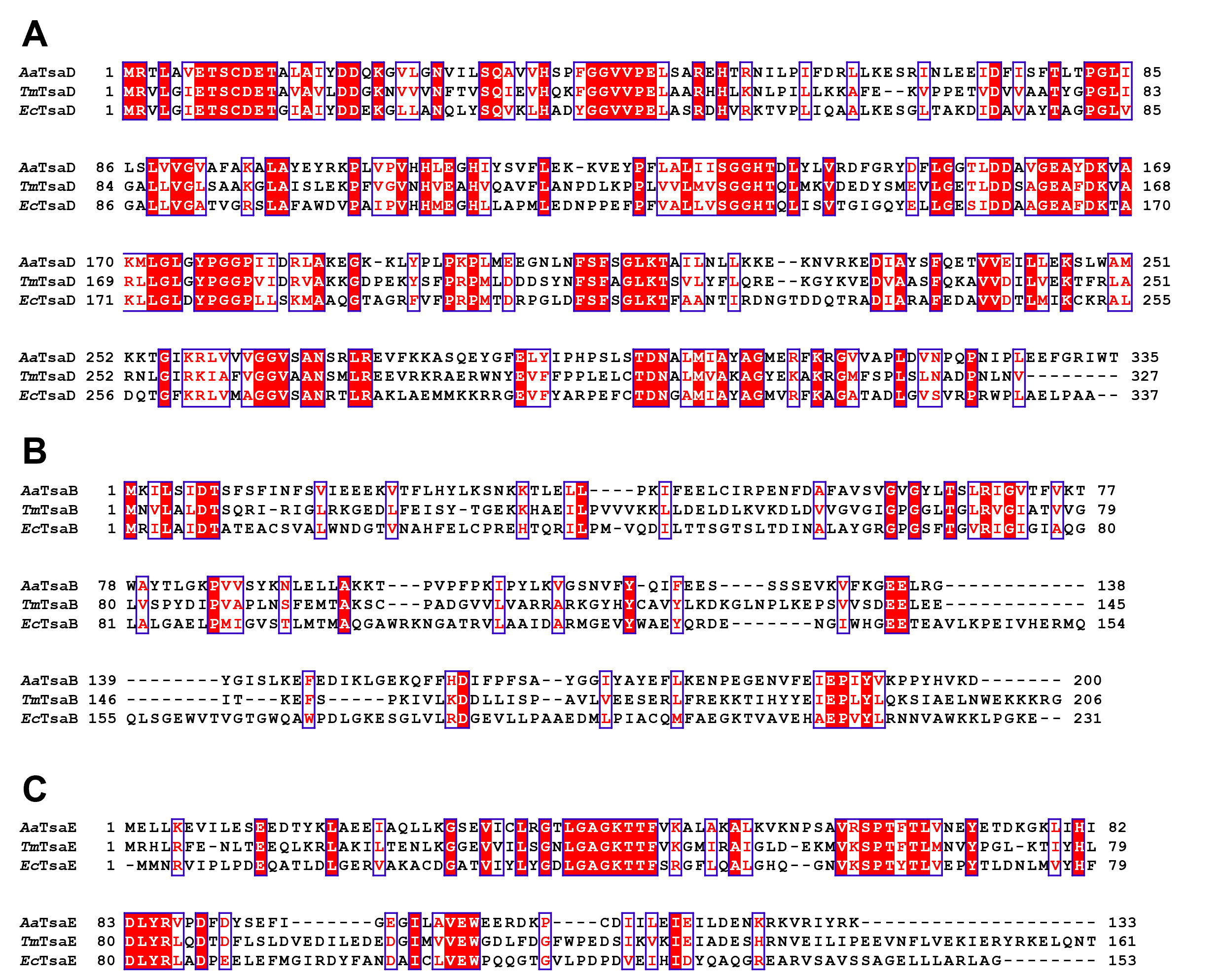


**Supplementary Figure 1.** Multiple sequence alignment of TsaD, TsaB and TsaE proteins from *A. aeolicus* (*Aa*), *T. maritima* (*Tm*) and *E. coli* (*Ec*). Strictly conserved residues are highlighted by a red background. **(A)** *Aa*TsaD (Uniprot: O66986) aligned with *Tm*TsaD (Uniprot: Q9WXZ2) and *Ec*TsaD (Uniprot: P05852). **(B)** *Aa*TsaB (Uniprot: O66494) aligned with *Tm*TsaB (Uniprot: Q9WZX7) and *Ec*TsaB (Uniprot: P76256). **(C)** *Aa*TsaE (Uniprot: O67011) aligned with *Tm*TsaE (Uniprot: Q9X1W7) and *Ec*TsaE (Uniprot: P0AF67).


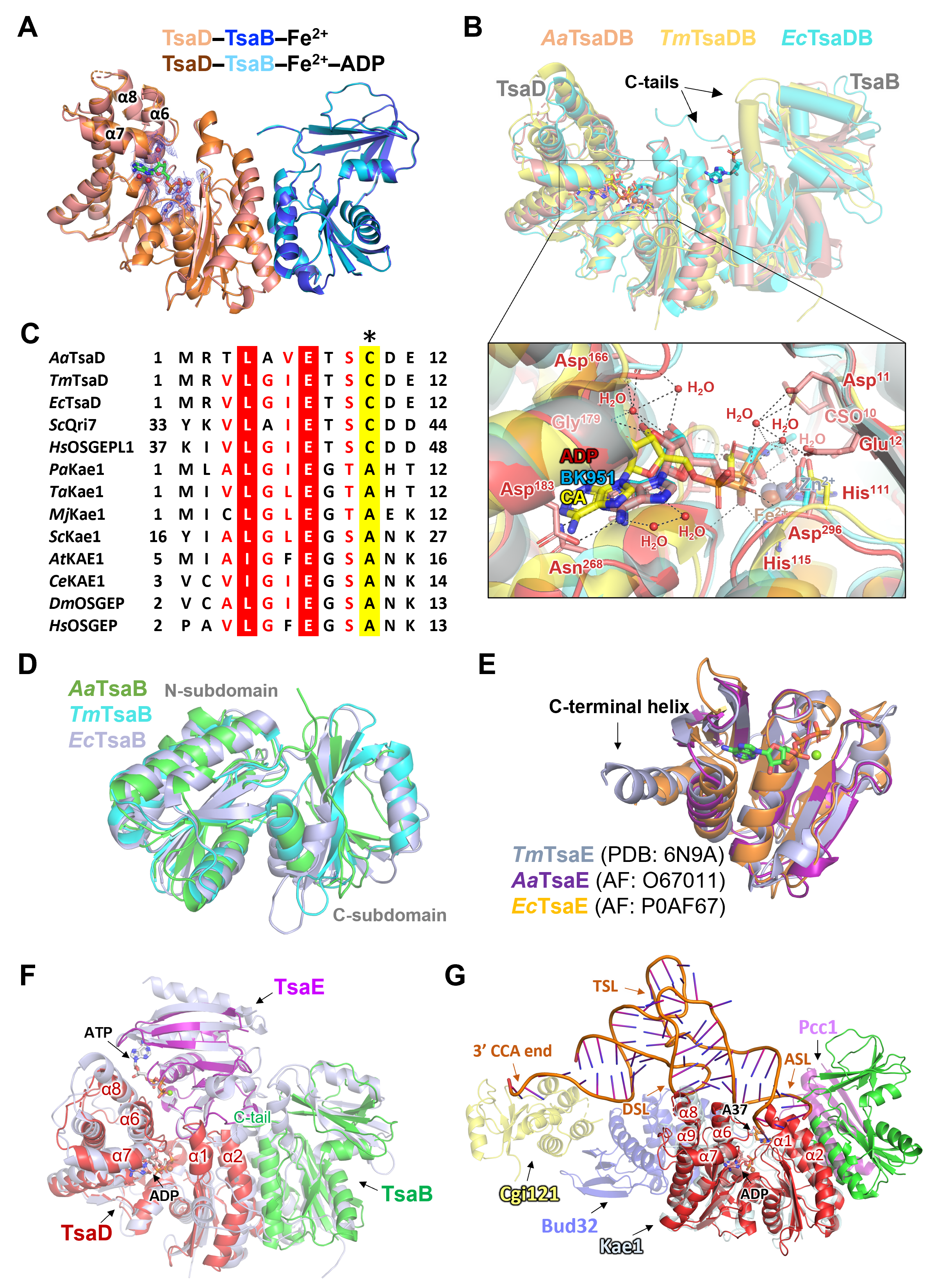


**Supplementary Figure 2.** Structural comparison of TsaD, TsaB and TsaE. **(A)** Structural alignment of *Aa*TsaD–TsaB–Fe^2+^ and *Aa*TsaD–TsaB–Fe^2+^–ADP manifests that ADP induces conformational changes of α6, α7 and α8 of TsaD. **(B)** Structural juxtaposition of *Aa*TsaD–TsaB–ADP–Fe^2+^, *Tm*TsaD–TsaB–CA–Zn^2+^ (PDB: 6N9A) and *Ec*TsaD–TsaB–BK951–Zn^2+^ (PDB: 6Z81). The zoomed region shows a close-up view of the bonding network of ADP in the catalytic site of *Aa*TsaD and overlaid ligands in the catalytic sites of TsaDs. **(C)** Local sequence alignment of *Aa*TsaD, *Tm*TsaD, *Ec*TsaD, *Sc*Qri7, *Hs*OSGEPL1, *Pa*Kae1, *Ta*Kae1, *Mj*Kae1, *Sc*Kae1, *At*KAE1, *Ce*KAE1, *Dm*OSGEP and *Hs*OSGEP. **(D)** Structural juxtaposition of *Aa*TsaB, *Tm*TsaB (PDB: 2A6A) and *Ec*TsaB (PDB: 1OKJ). **(E)** Structural juxtaposition of *Aa*TsaE (AF ID: O67011), *Tm*TsaE (PDB: 6N9A) and *Ec*TsaE (AF ID: P0AF67). **(F)** Structural alignment of *A. aeolicus* TsaDB and TsaE (AF prediction model) to *T. maritima* TsaDBE complex (PDB: 6N9A, colored in gray). **(G)** Structural alignment of *A. aeolicus* TsaD to Kae1 in archaean KEOPS–tRNA^Lys^_UUU_ complex. KEOPS subunits and D stem loop (DSL), anticodon stem loop (ASL), TΨC stem loop (TSL) and 3′ CCA end of tRNA are labeled. *Sc*, *S. cerevisiae*; *Hs*, *H. sapiens*; *Pa*, *P. abyssi*; *Ta*, *T. acidophilum*; *Mj*, *M. jannaschii*; *At*, *A. thaliana*; *Ce*, *C. elegans*; *Dm*, *D. melanogaster*.


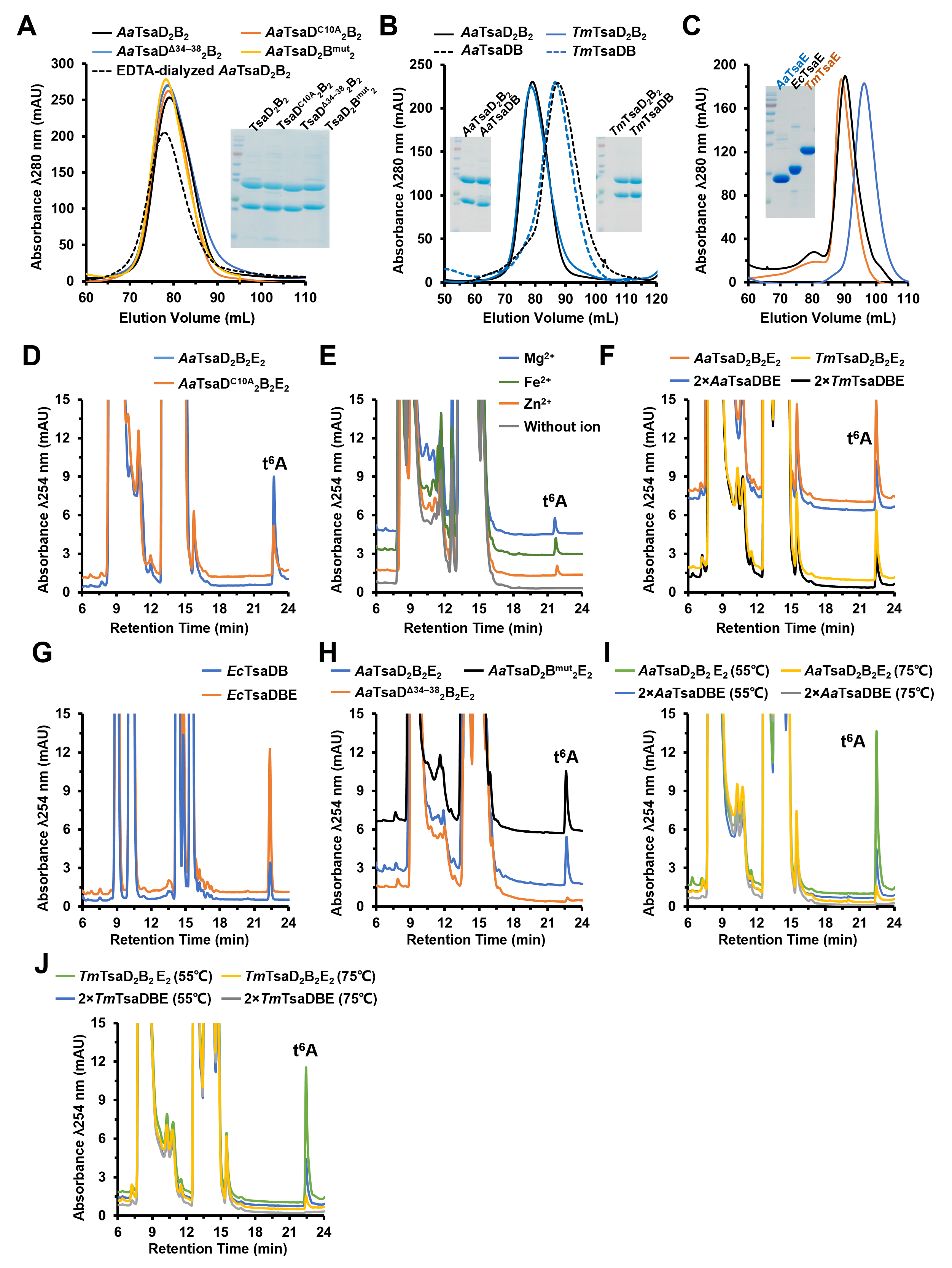


**Supplementary Figure 3.** Purification and biochemical properties of TsaD–TsaB and TsaE from *A. aeolicus*, *T. maritima* and *E. coli*. **(A)** Size-exclusion chromatography (SEC) profiles and SDS–PAGE analysis of *Aa*TsaD_2_B_2_ and mutants. **(B)** SEC profiles and SDS–PAGE analysis of *Aa*TsaD_2_B_2_, *Aa*TsaDB, *Tm*TsaD_2_B_2_ and *Tm*TsaDB. **(C)** SEC profiles and SDS–PAGE analysis of TsaEs from *A. aeolicus*, *T. maritima* and *E. coli*. **(D)**–**(I)** LC–MS analysis of tRNA t^6^A formation in assays contained enzymes and tRNAs as the following. **(D)** 5 μM *Aa*TsaC, 5 μM *Aa*TsaD_2_B_2_E_2_ or *Aa*TsaD^C10A^_2_B_2_E_2_ and 60 μM *in vitro* transcribed (IVT) *Aa*tRNA^Lys^_UUU_. **(E)** 5 µM EDTA-dialyzed *Aa*TsaD_2_B_2_, 30 µM isolated TC-AMP, 60 μM metal-depleted IVT *Aa*tRNA^Lys^_UUU_ and 100 μM Mg^2+^, Fe^2+^ or Zn^2+^. **(F)** 5 μM *Aa*TsaC, 5 μM *Aa*TsaD_2_B_2_E_2_ or 10 μM *Aa*TsaDBE and 60 μM IVT *Aa*tRNA^Lys^_UUU_; 5 μM *Aa*TsaC, 5 μM *Tm*TsaD_2_B_2_E_2_ or 10 μM *Tm*TsaDBE and 60 μM IVT *Tm*tRNA^Lys^_UUU_. **(G)** 5 μM *Aa*TsaC, 5 μM *Ec*TsaDB or *Ec*TsaDBE and 60 μM IVT *Ec*tRNA^Lys^_UUU_. **(H)** 5 μM *Aa*TsaC, 5 μM *Aa*TsaD_2_B_2_E_2_ or mutants and 60 μM IVT *Aa*tRNA^Lys^_UUU_. **(I)** 5 μM *Aa*TsaC, 5 μM *Aa*TsaD_2_B_2_E_2_ or 10 μM *Aa*TsaDBE and 60 μM IVT *Aa*tRNA^Lys^_UUU_ at 55°C or 75°C. **(J)** 5 μM *Aa*TsaC, 5 μM *Tm*TsaD_2_B_2_E_2_ or 10 μM *Tm*TsaDBE and 60 μM IVT *Tm*tRNA^Lys^_UUU_ at 55°C or 75°C. The HPLC areas of nucleosides were integrated and normalized for qualification of the t^6^A modification efficiency.


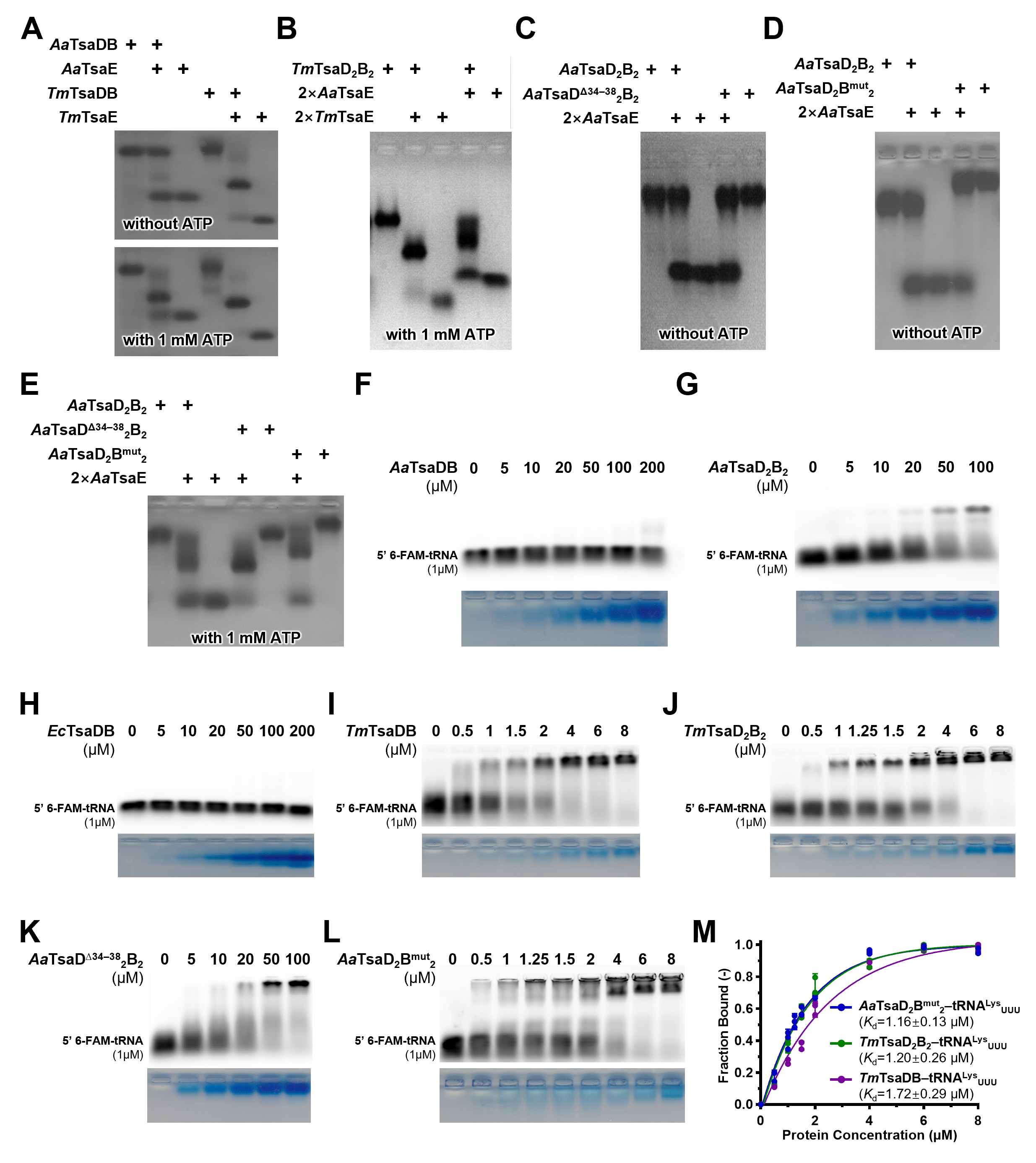


**Supplementary Figure 4.** Interaction analysis between proteins and proteins and between proteins and tRNAs. **(A)** Native gel shift analysis of interactions between TsaDB and TsaE from *A. aeolicus* and *T. maritima* in the absence of or in the presence of 1 mM ATP and 2 mM MgCl_2_. **(B)** Native gel shift analysis of interactions between *Tm*TsaD_2_B_2_ and two-fold *Aa*TsaE or *Tm*TsaE (in molar concentration) in the presence of 1 mM ATP and 2 mM MgCl_2_. **(C)** Native gel shift analysis of interactions between *A. aeolicus* TsaD_2_B_2_ or TsaD^Δ34–38^_2_B_2_ and two-fold TsaE in the absence of ATP and MgCl_2_. **(D)** Native gel shift analysis of interactions between *A. aeolicus* TsaD_2_B_2_ or TsaD_2_B^mut^_2_ and two-fold TsaE in the absence of ATP and MgCl_2_. **(E)** Native gel shift analysis of the interactions between *A. aeolicus* TsaD_2_B_2_, TsaD^Δ34–38^_2_B_2_ or TsaD_2_B^mut^_2_ and two-fold TsaE in the presence of 1 mM ATP and 2 mM MgCl_2_. **(F)**–**(L)** Representative gels show EMSA analysis of the interaction between 1 μM 5’ 6-FAM-tRNA^Lys^_UUU_ and proteins as the following. The upper panels show the migration of tRNAs and the lower panels show the migration of proteins. **(F)** 5’ 6-FAM-*Aa*tRNA^Lys^_UUU_ and *Aa*TsaDB. **(G)** 5’ 6-FAM-*Aa*tRNA^Lys^_UUU_ and *Aa*TsaD_2_B_2_. **(H)** 5’ 6-FAM-*Aa*tRNA^Lys^_UUU_ and *Ec*TsaDB. **(I)** 5’ 6-FAM-*Tm*tRNA^Lys^_UUU_ and *Tm*TsaDB. **(J)** 5’ 6-FAM-*Tm*tRNA^Lys^_UUU_ and *Tm*TsaD_2_B_2_. **(K)** 5’ 6-FAM-*Aa*tRNA^Lys^_UUU_ and *Aa*TsaD^Δ34–38^_2_B_2_. **(L)** 5’ 6-FAM-*Aa*tRNA^Lys^_UUU_ and *Aa*TsaD_2_B^mut^_2_. **(M)** Plot of fraction of tRNA bound quantified by densitometry as the ratio of upper-band intensity to total intensity in each lane as measured by EMSA. Error bars represent standard deviations from triplicate measurements.


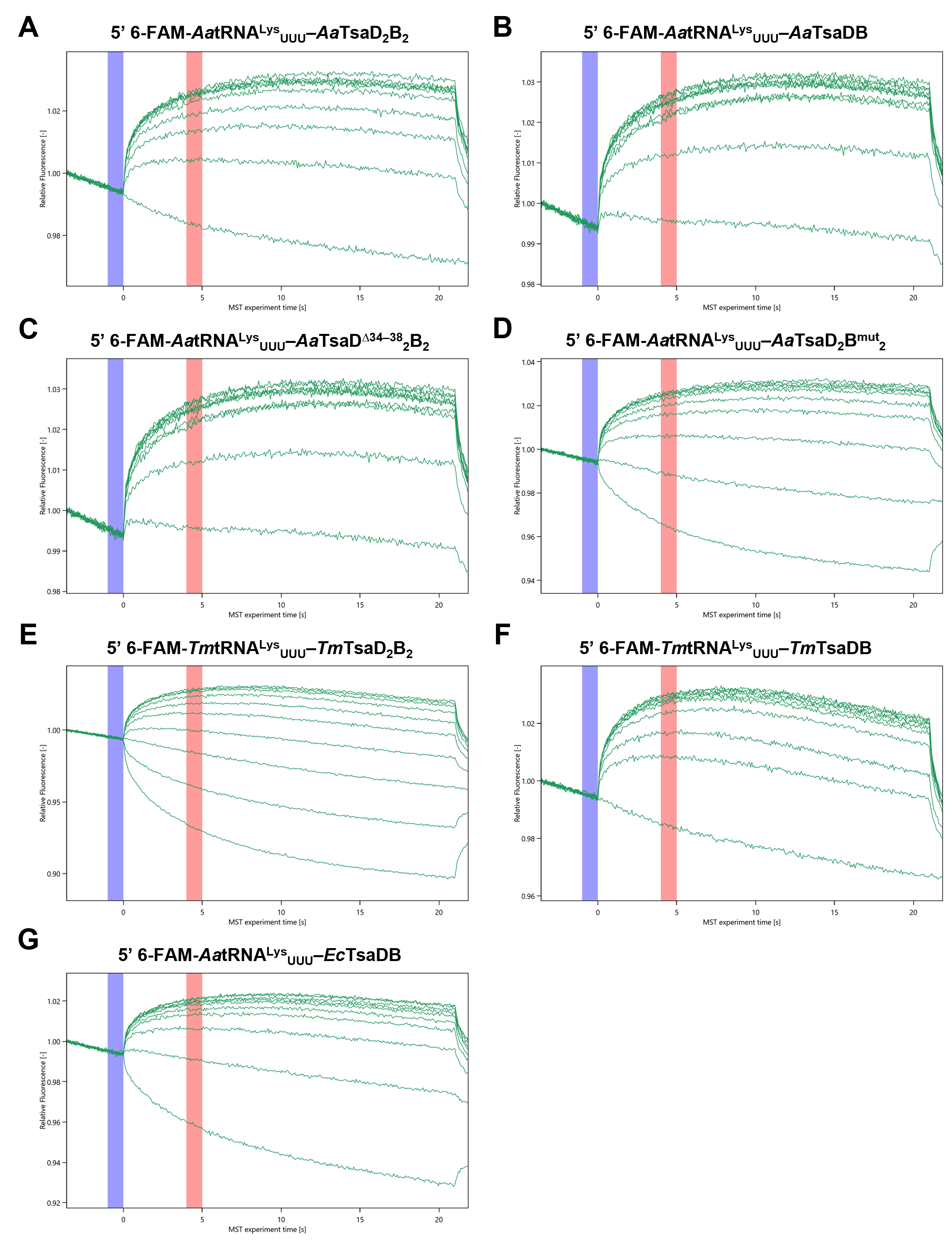


**Supplementary Figure 5.** Raw microscale thermophoresis (MST) traces are shown as representative examples between 10 nM 5’ 6-FAM-tRNA^Lys^_UUU_ and 195 nM–100 μM proteins as the following. **(A)** 5’ 6-FAM-*Aa*tRNA^Lys^_UUU_ and *Aa*TsaD_2_B_2_. **(B)** 5’ 6-FAM-*Aa*tRNA^Lys^_UUU_ and *Aa*TsaDB. **(C)** 5’ 6-FAM-*Aa*tRNA^Lys^_UUU_ and *Aa*TsaD^Δ34–38^_2_B_2_. **(D)** 5’ 6-FAM-*Aa*tRNA^Lys^_UUU_ and *Aa*TsaD_2_B^mut^_2_. **(E)** 5’ 6-FAM-*Tm*tRNA^Lys^_UUU_ and *Tm*TsaD_2_B_2_. **(F)** 5’ 6-FAM-*Tm*tRNA^Lys^_UUU_ and *Tm*TsaDB. **(G)** 5’ 6-FAM-*Aa*tRNA^Lys^_UUU_ and *Ec*TsaDB. Blue and red bars indicate the beginning and ending of the time period used to calculate normalized rates of reduction in the fluorescence for capillaries over a 20-seconds-long laser-induced temperature jump. Each curve plots the fall in fluorescence in a separate capillary, and the subsequent rise, over 1 s, after heating is switched off.


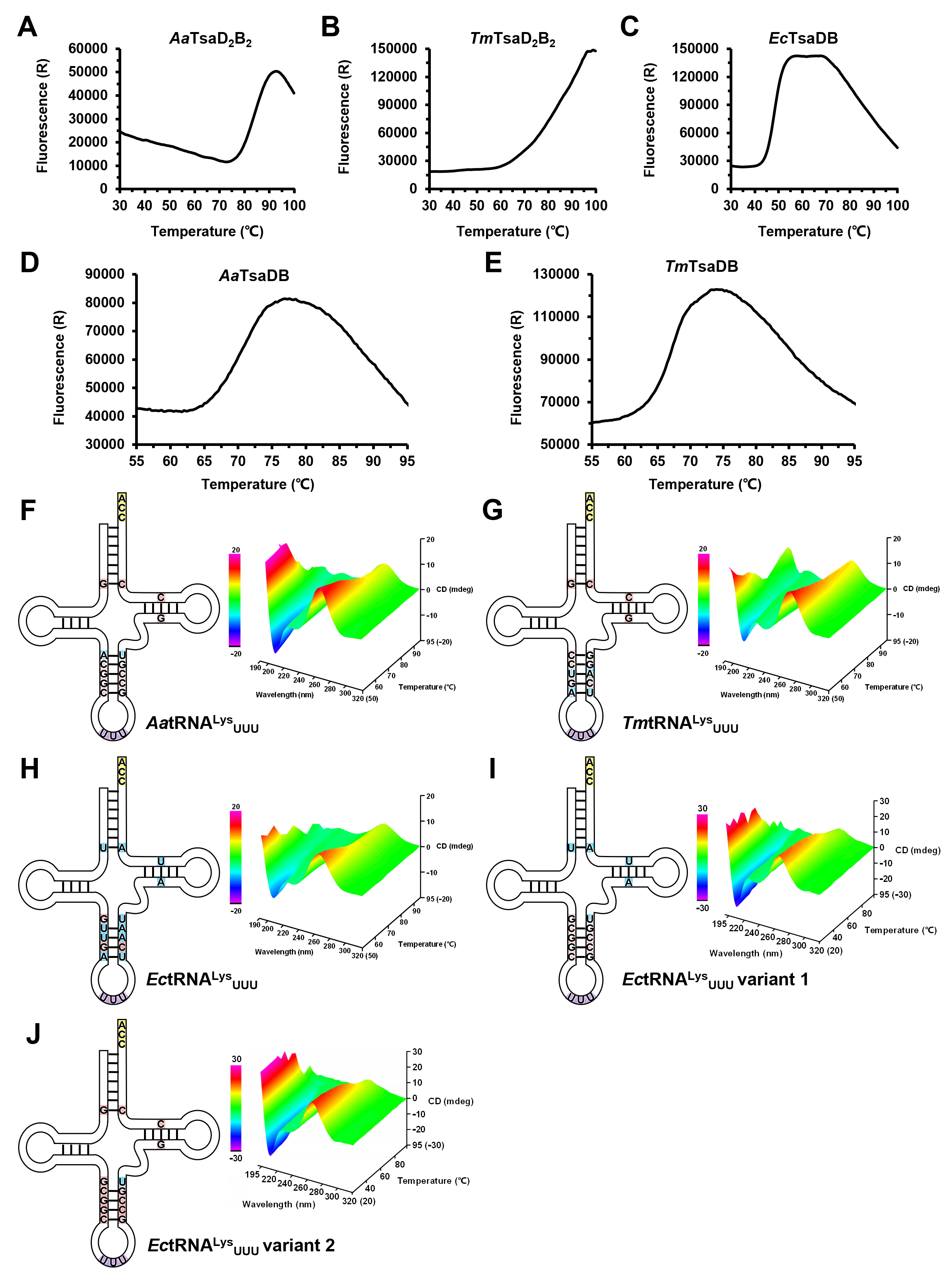


**Supplementary Figure 6.** Thermostability analysis of proteins by thermal shift assay and folding analysis of tRNAs by CD spectra. **(A)**–**(E)** Representative melt curves of *Aa*TsaD_2_B_2_ **(A)**, *Tm*TsaD_2_B_2_ **(B)**, *Ec*TsaDB **(C)**, *Aa*TsaDB **(D)** and *Tm*TsaDB **(E)**. **(F)**–**(J)** The predicted structure (left panel) and melting CD spectra (right panel) of *Aa*tRNA^Lys^_UUU_ **(F)**, *Tm*tRNA^Lys^_UUU_ **(G)**, *Ec*tRNA^Lys^_UUU_ **(H)**, *Ec*tRNA^Lys^_UUU_ variant 1 **(I)** and *Ec*tRNA^Lys^_UUU_ variant 2 **(J)**. Melting temperature (*T*_m_) was derived from these curves according to standard protocols.
